# Supplementary material for: Biomimetic selenocystine based dynamic combinatorial chemistry for thiol-disulfide exchange
Source: Nat Commun. 2021 Jan 8;12:163. doi: 10.1038/s41467-020-20415-6 (PMC7794297; doi:10.1038/s41467-020-20415-6)
Supplement: Supplementary file 1 — Supplementary Information [file 41467_2020_20415_MOESM1_ESM.pdf]

SUPPLEMENTARY INFORMATION

Biomimetic selenocystine based dynamic combinatorial chemistry for thiol-disulfide exchange

Andrea Canal-Martín, Ruth Pérez-Fernández*

Structural and Chemical biology department, Centro de Investigaciones Biológicas "Margarita Salas", CIB-CSIC, Madrid 28040, Spain.

OUTLINE

1. **SUPPLEMENTARY FIGURES 2**

2. **SUPPLEMENTARY TABLES 30**

3. **SUPPLEMENTARY METHODS..37**

4. **SUPPLEMENTARY DISCUSSION...40**

5. **SUPPLEMENTARY REFERENCES 42**

1. SUPPLEMENTARY FIGURES

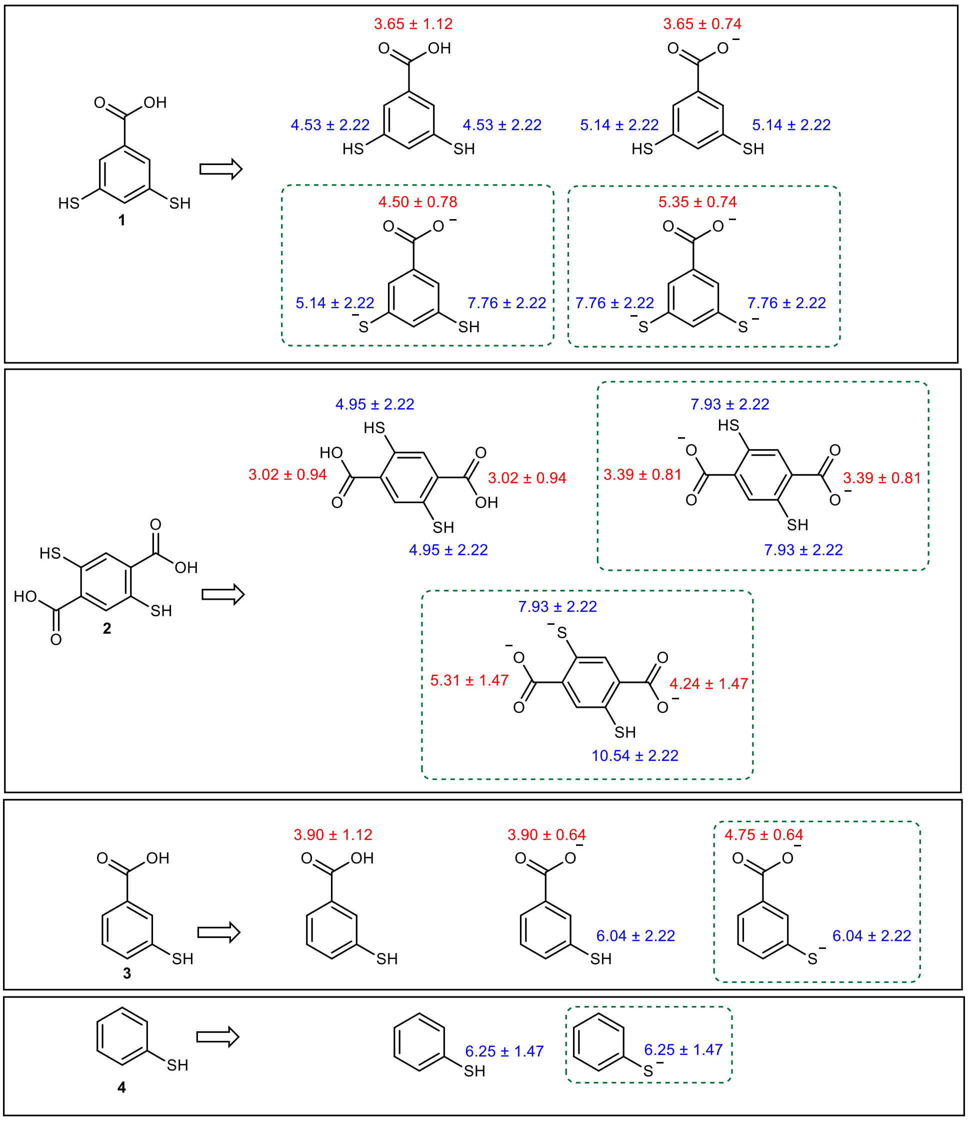

Supplementary Figure 1. pK_a values of building blocks **1-4** calculated with Epik as implemented in Schrödinger Suite Release 2020-2.[1] The most abundant species at pH 7.8 are framed in a green dashed box.

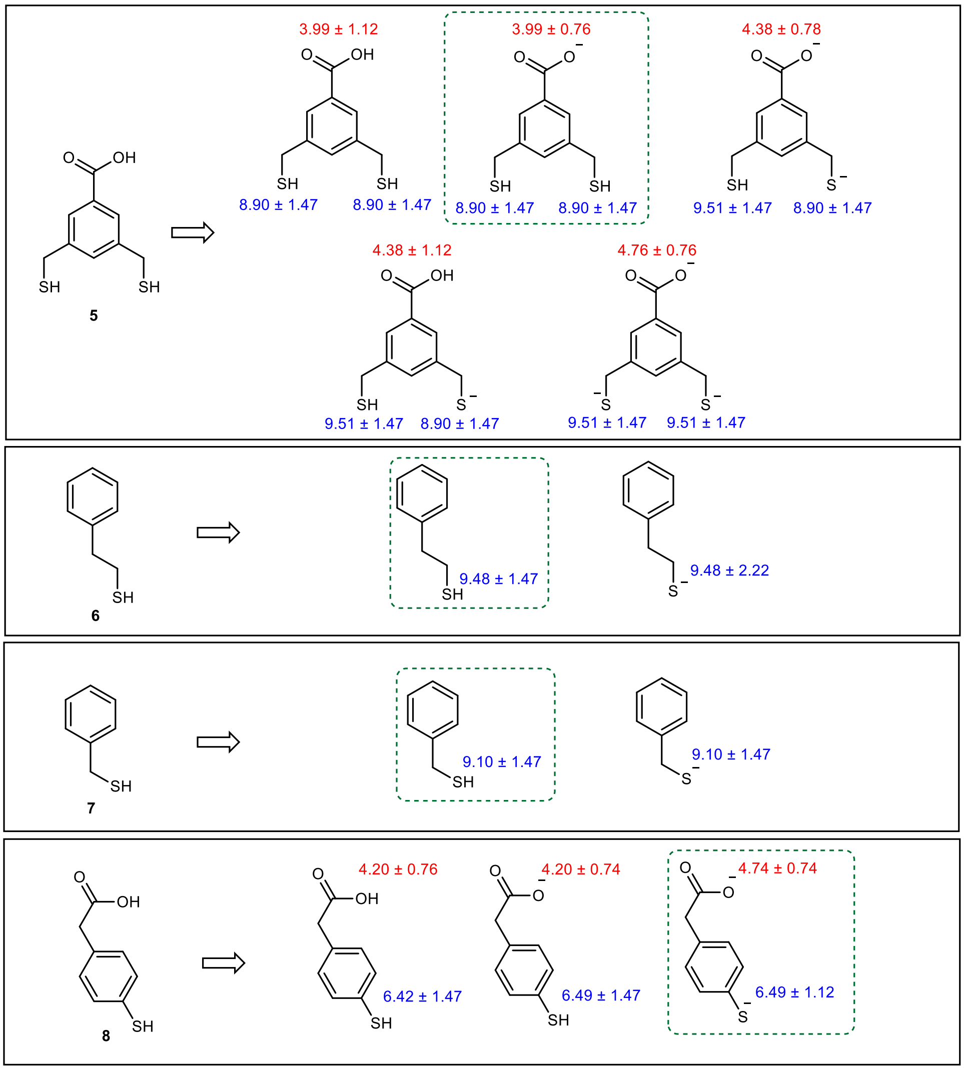

Supplementary Figure 2. pK_a values of building blocks **5-8** calculated with Epik as implemented in Schrödinger Suite Release 2020-2.[1] The most abundant species at pH 7.8 are framed in a green dashed box.

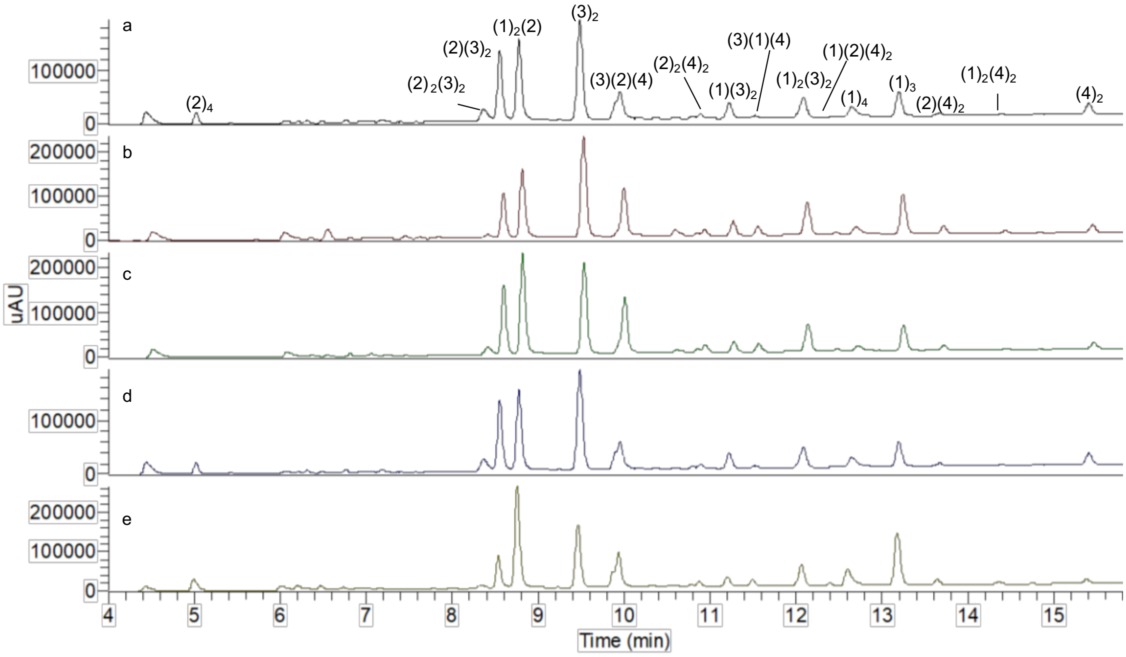

Supplementary Figure 3. Chromatograms of the DCL performed in different conditions. **a**, In absence of any catalyst (black). **b**, In presence of Cys_{ox} at 10 % mol in Tris 20 mM buffer pH 7.8 for 72 h (red). **c,** In presence of Cys_{ox} at 10 % mol in Tris 20 mM buffer pH 8.8 for 72 h (green). **d**, In presence of Sec_{ox} at 10 % mol in Tris 20 mM buffer pH 7.8 for 24 h (blue). **e**, In presence of Sec_{ox} at 10 % mol in Tris 20 mM buffer pH 8.8 for 24 h (yellow). Although Cys_{ox} is deprotonated at pH 8.8, the equilibration time for the DCL is not altered. Experiments were performed in triplicate and repeated three times with similar results.

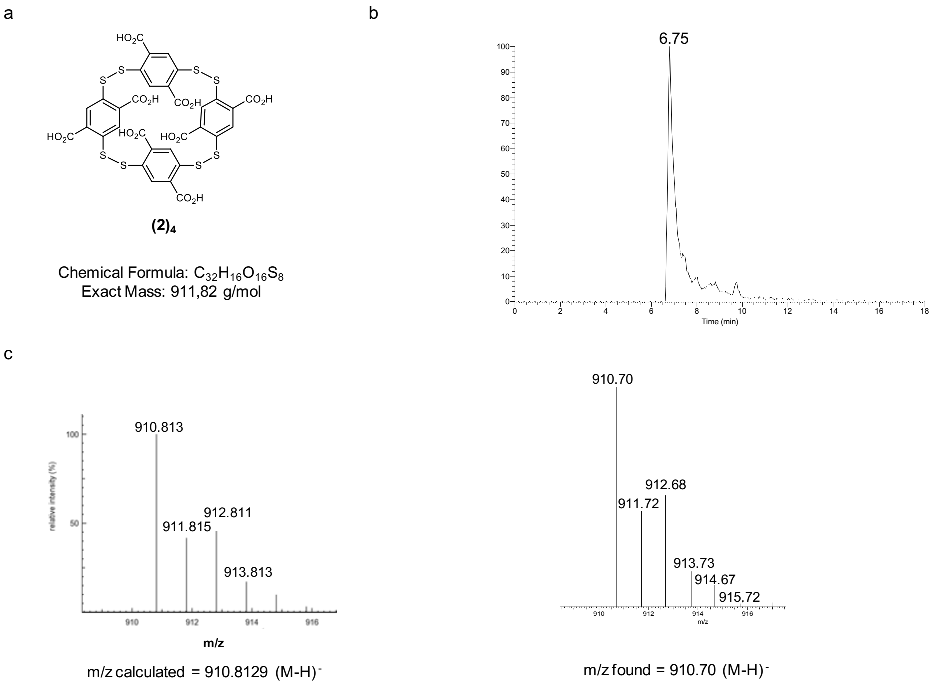

Supplementary Figure 4. a, Structure. **b**, Retention time analyzed by HPLC. **c**, Calculated isotope pattern and found isotope pattern corresponding to $[M\text{-}H]^-$ of (**2**)$_4$.

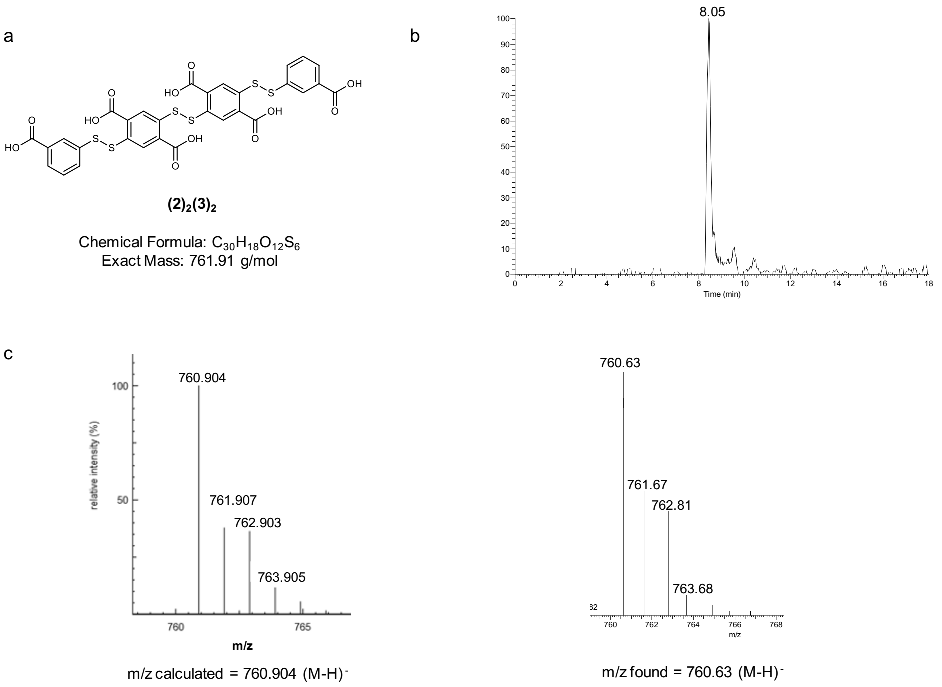

Supplementary Figure 5. **a**, Structure. **b**, Retention time analyzed by HPLC. **c**, Calculated isotope pattern and found isotope pattern corresponding to [M-H]$^-$ of (**2**)$_2$(**3**)$_2$.

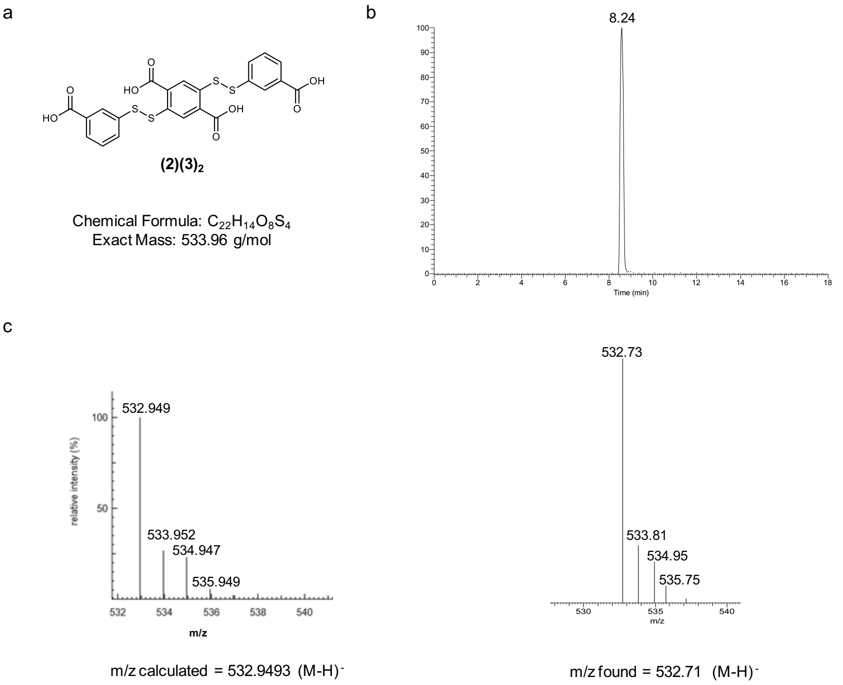

Supplementary Figure 6. **a**, Structure. **b**, Retention time analyzed by HPLC. **c**, Calculated isotope pattern and found isotope pattern corresponding to [M-H]$^-$ of (**2**)(**3**)$_2$.

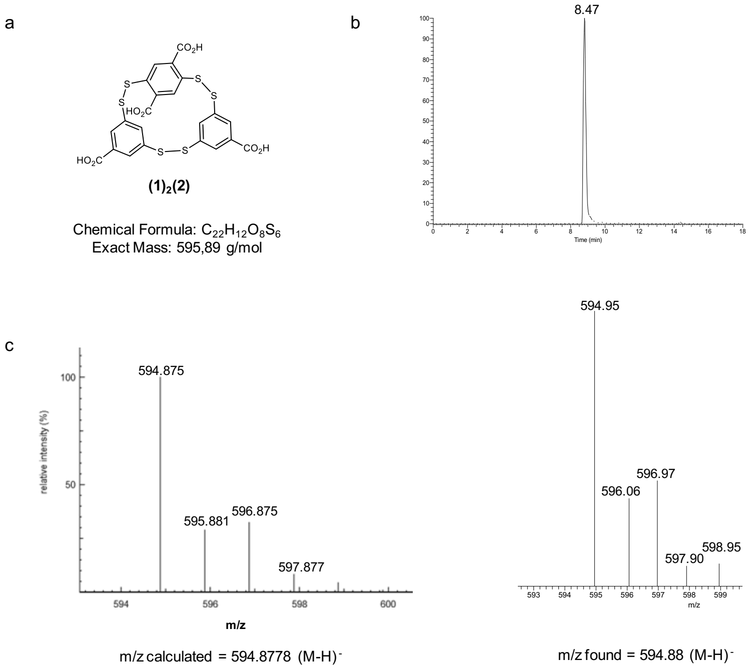

Supplementary Figure 7. **a**, Structure. **b**, Retention time analyzed by HPLC. **c**, Calculated isotope pattern and found isotope pattern corresponding to $[M\text{-}H]^-$ of (**1**)$_2$(**2**).

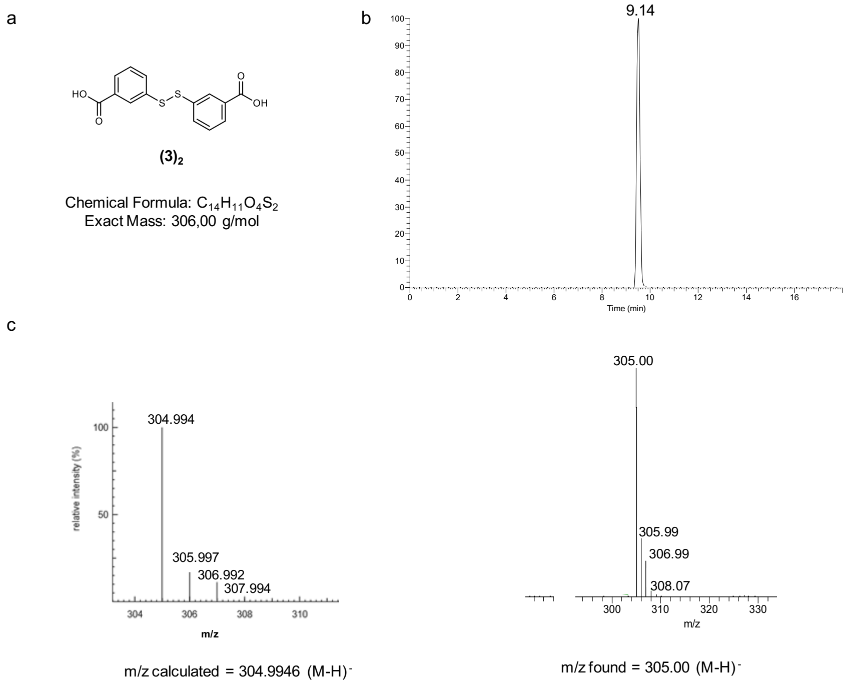

Supplementary Figure 8. **a**, Structure. **b**, Retention time analyzed by HPLC. **c**, Calculated isotope pattern and found isotope pattern corresponding to $[M\text{-}H]^-$ of (**3**)$_2$.

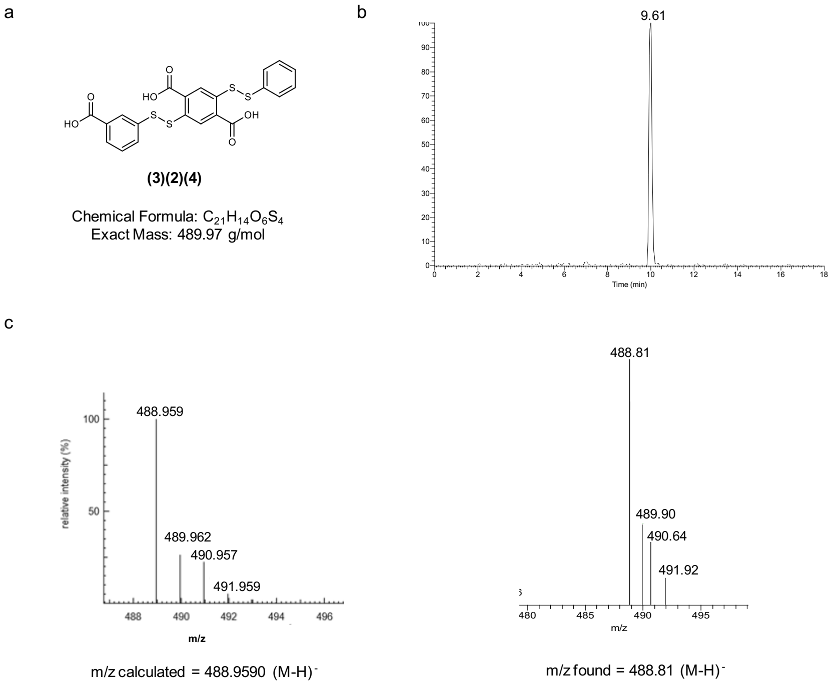

Supplementary Figure 9. a, Structure. **b**, Retention time analyzed by HPLC. **c**, Calculated isotope pattern and found isotope pattern corresponding to [M-H]$^-$ of (**3**)(**2**)(**4**) .

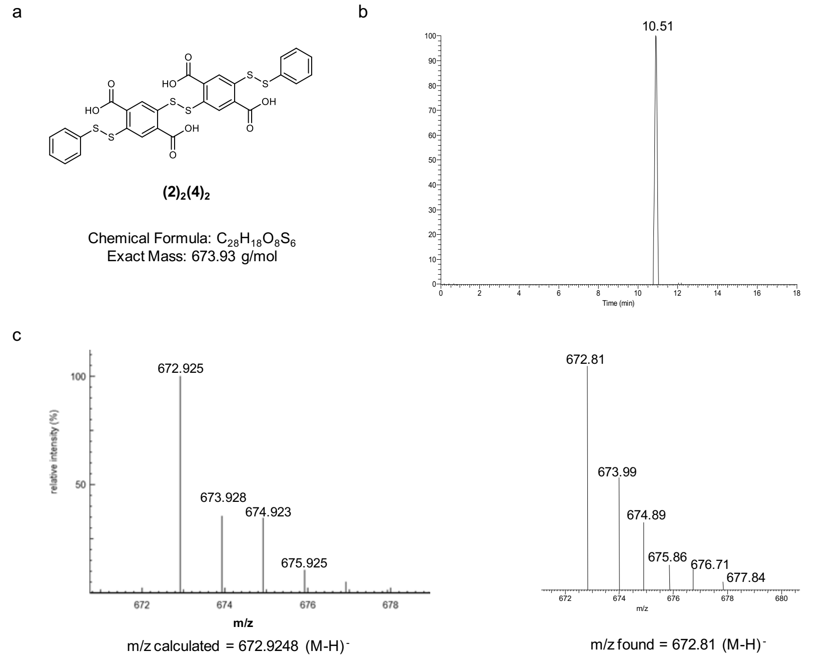

Supplementary Figure 10. a, Structure. **b**, Retention time analyzed by HPLC. **c**, Calculated isotope pattern and found isotope pattern corresponding to [M-H]$^-$ of (**2**)$_2$(**4**)$_2$.

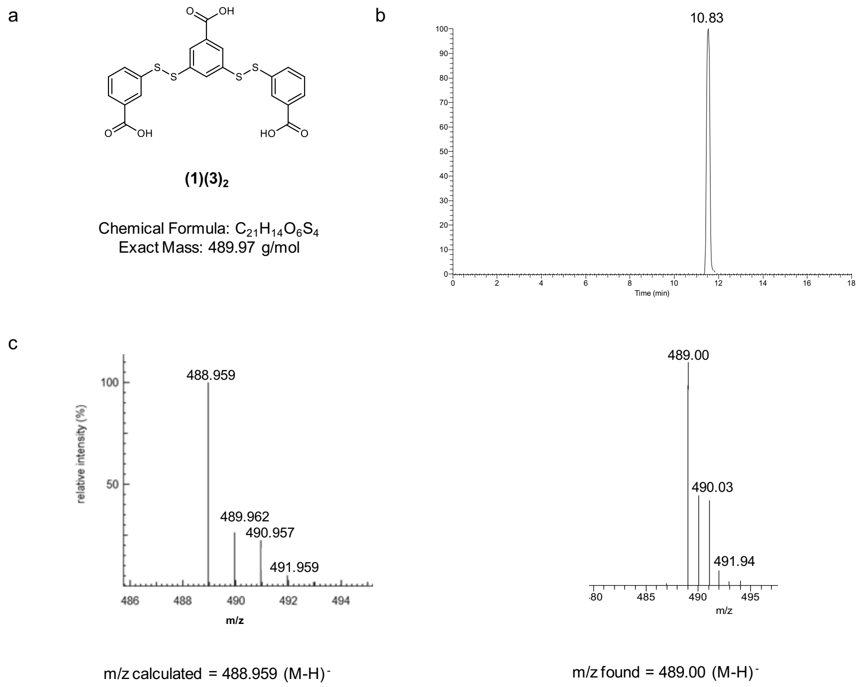

Supplementary Figure 11. **a**, Structure. **b**, Retention time analyzed by HPLC. **c**, Calculated isotope pattern and found isotope pattern corresponding to [M-H]$^-$ of (**1**)(**3**)$_2$.

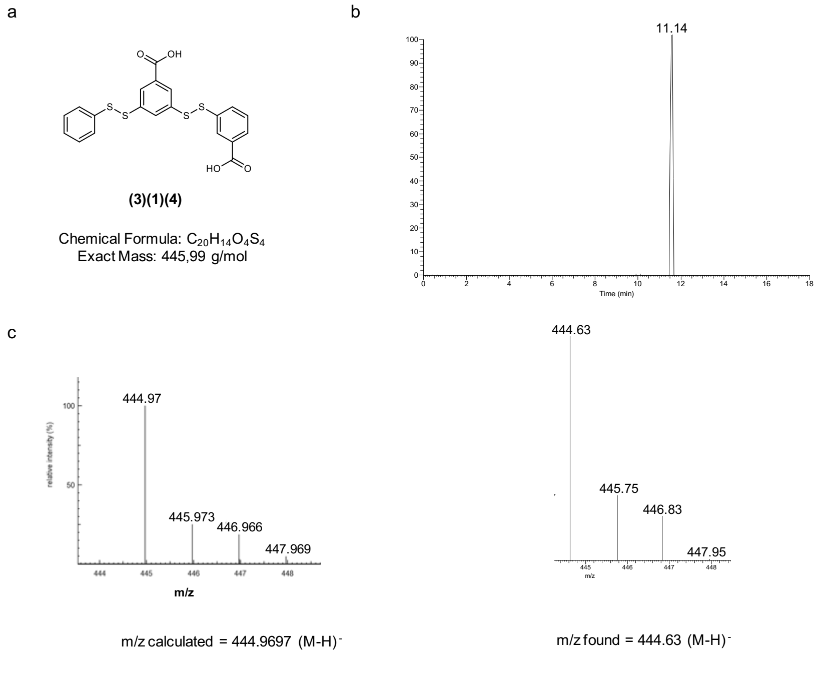

Supplementary Figure 12. **a**, Structure. **b**, Retention time analyzed by HPLC. **c**, Calculated isotope pattern and found isotope pattern corresponding to [M-H]$^-$ of (**3**)(**1**)(**4**).

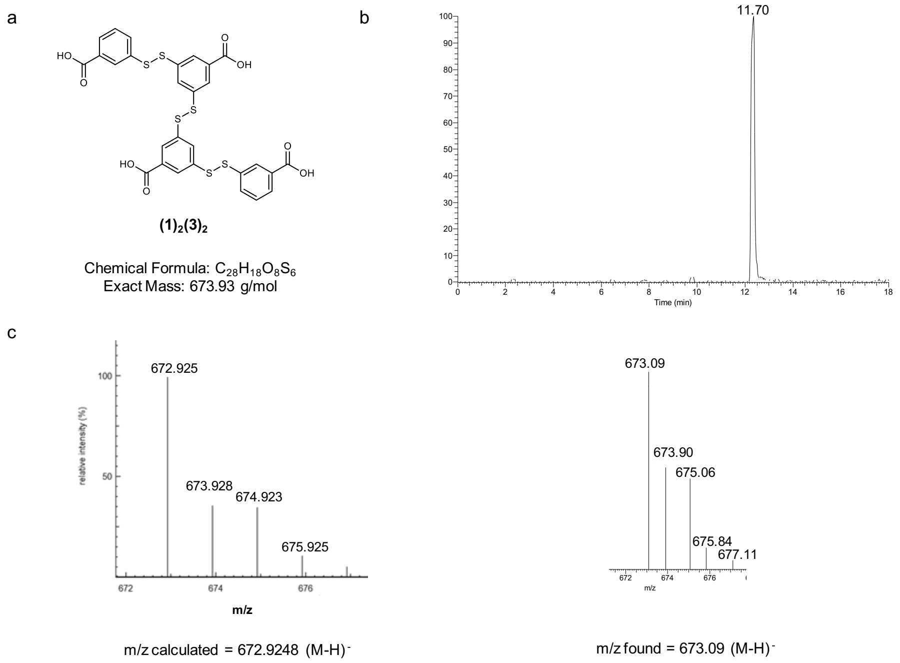

Supplementary Figure 13. **a**, Structure. **b**, Retention time analyzed by HPLC. **c**, Calculated isotope pattern and found isotope pattern corresponding to $[M\text{-}H]^-$ of $(\mathbf{1})_2(\mathbf{3})_2$.

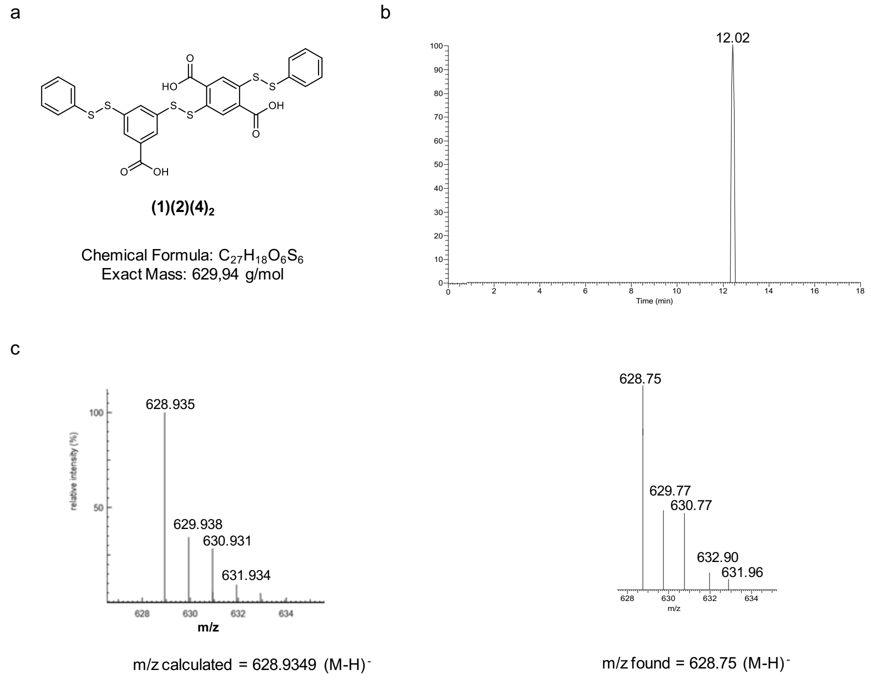

Supplementary Figure 14. **a**, Structure. **b**, Retention time analyzed by HPLC. **c**, Calculated isotope pattern and found isotope pattern corresponding to $[M\text{-}H]^-$ of $(\mathbf{1})(\mathbf{2})(\mathbf{4})_2$.

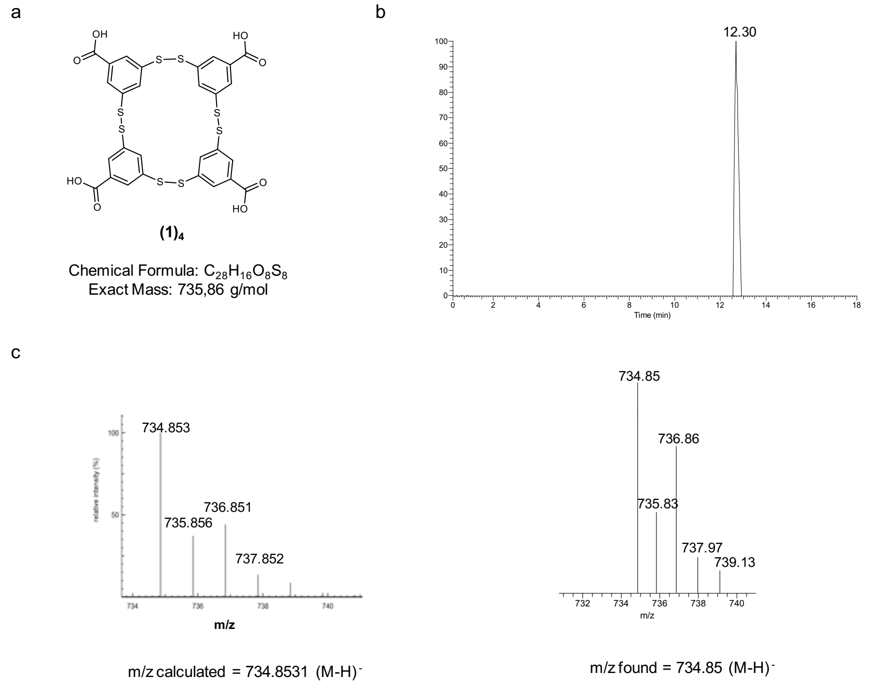

Supplementary Figure 15. **a**, Structure. **b**, Retention time analyzed by HPLC. **c**, Calculated isotope pattern and found isotope pattern corresponding to $[M\text{-}H]^-$ of (**1**)$_4$.

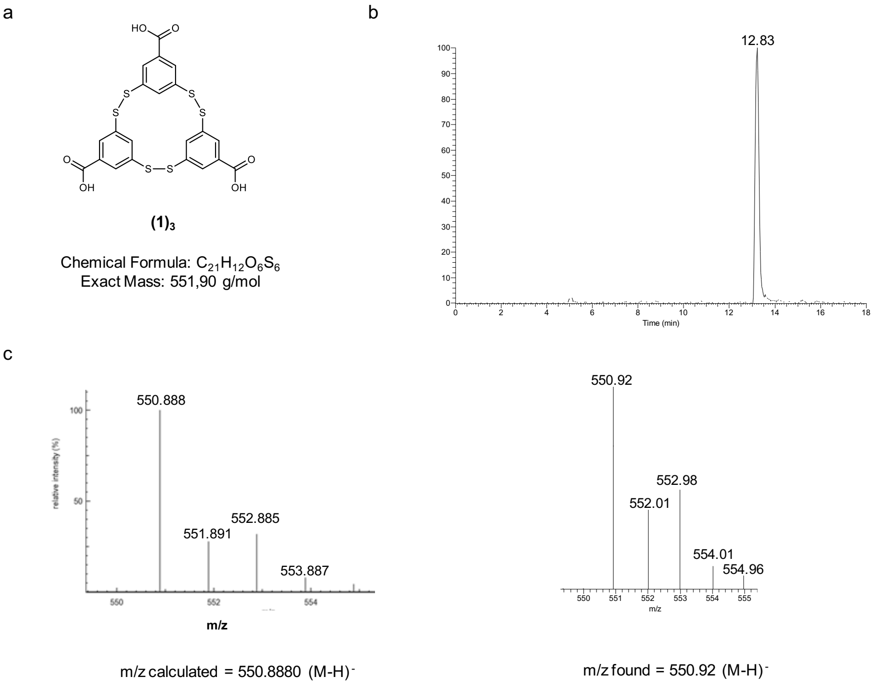

Supplementary Figure 16. **a**, Structure. **b**, Retention time analyzed by HPLC. **c**, Calculated isotope pattern and found isotope pattern corresponding to $[M\text{-}H]^-$ of (**1**)$_3$.

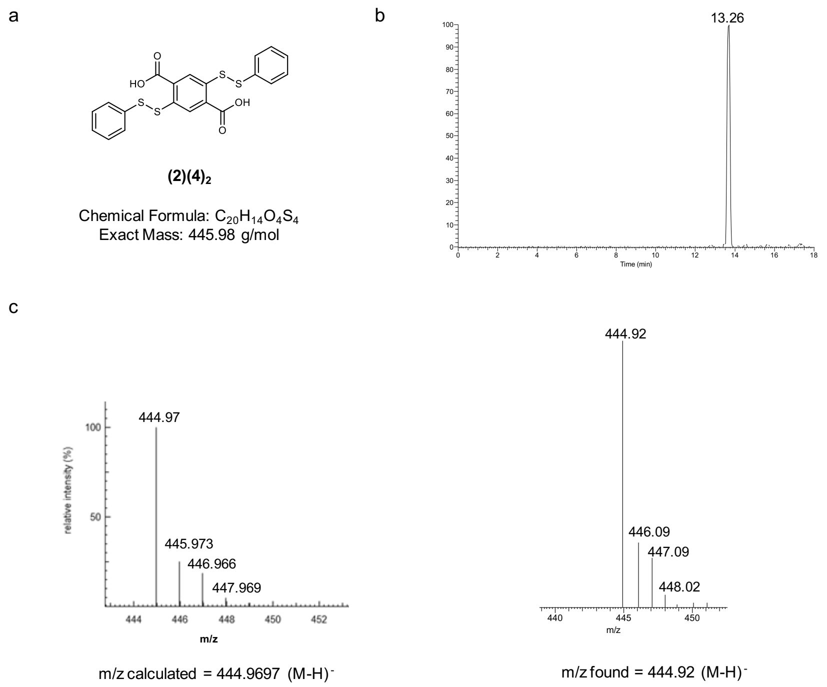

Supplementary Figure 17. a, Structure. **b**, Retention time analyzed by HPLC. **c**, Calculated isotope pattern and found isotope pattern corresponding to $[M\text{-}H]^-$ of (**2**)(**4**)$_2$.

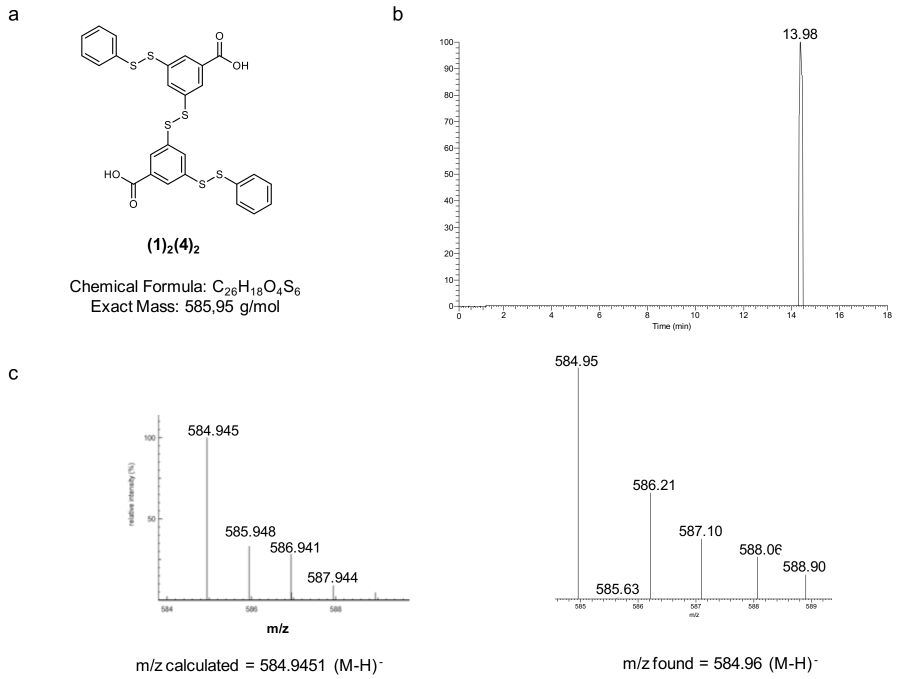

Supplementary Figure 18. a, Structure. **b**, Retention time analyzed by HPLC. **c**, Calculated isotope pattern and found isotope pattern corresponding to $[M\text{-}H]^-$ of (**1**)$_2$(**4**)$_2$.

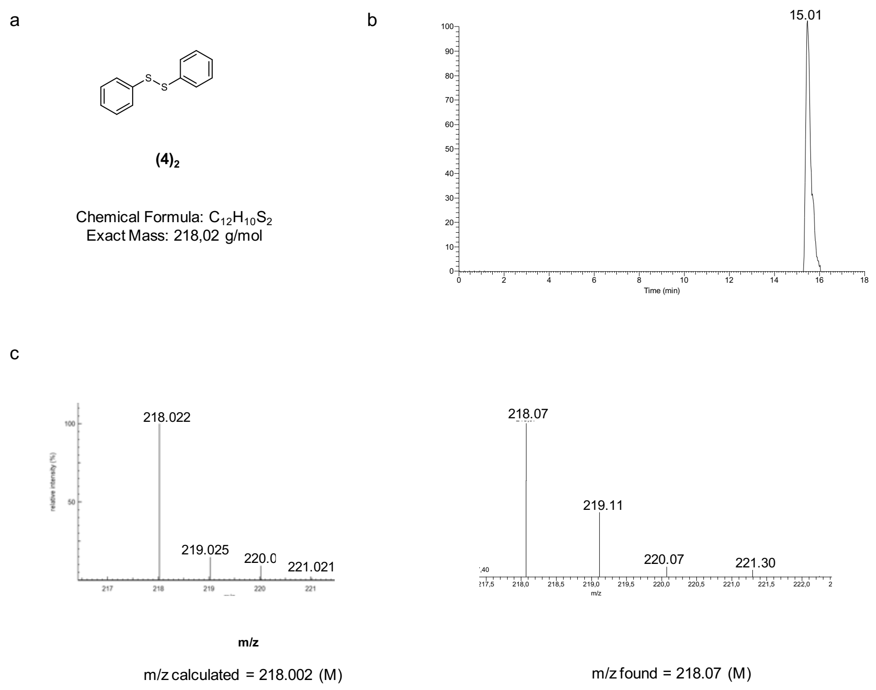

Supplementary Figure 19. **a**, Structure. **b**, Retention time analyzed by HPLC. **c**, Calculated isotope pattern and found isotope pattern corresponding to [M] of (**4**)$_2$.

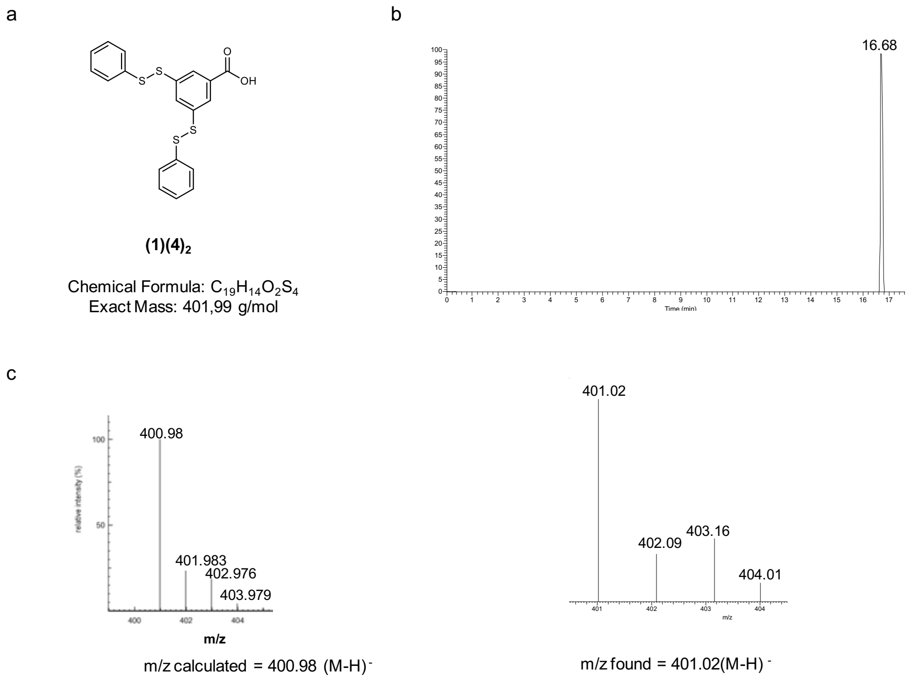

Supplementary Figure 20. **a**, Structure. **b**, Retention time analyzed by HPLC. **c**, Calculated isotope pattern and found isotope pattern corresponding to [M-H]$^-$ of (**1**)(**4**)$_2$.

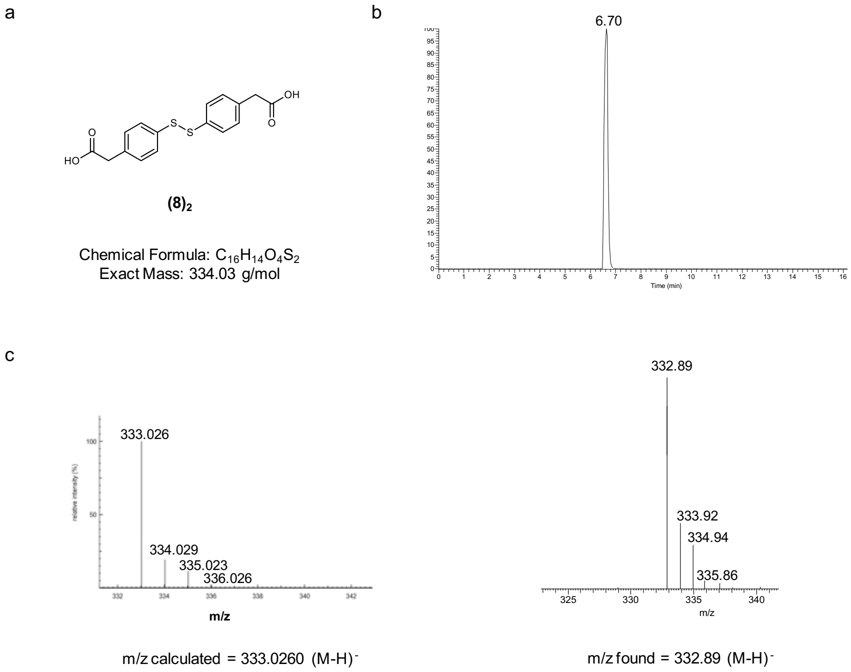

Supplementary Figure 21. a, Structure. **b**, Retention time analyzed by HPLC. **c**, Calculated isotope pattern and found isotope pattern corresponding to $[M\text{-}H]^-$ of (**8**)$_2$.

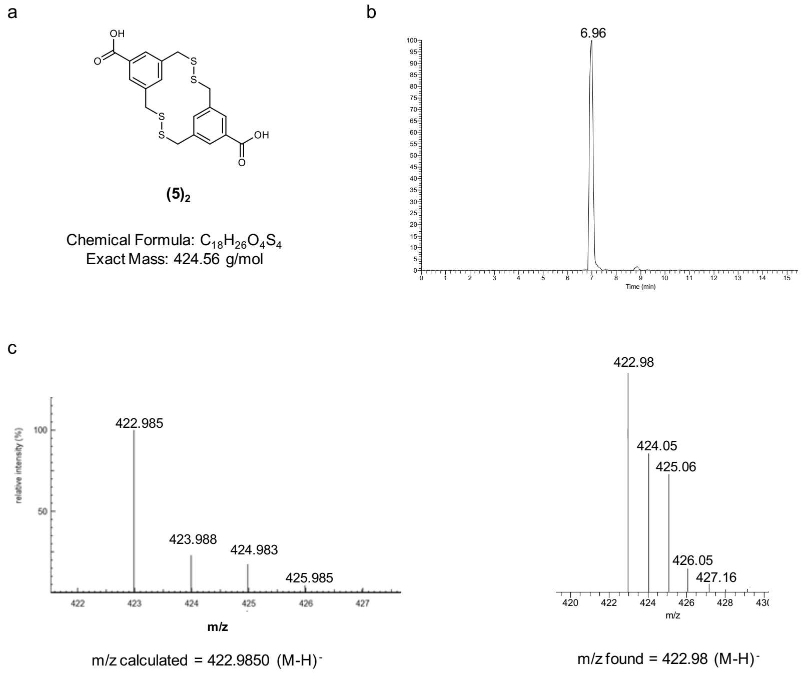

Supplementary Figure 22. a, Structure. **b**, Retention time analyzed by HPLC. **c**, Calculated isotope pattern and found isotope pattern corresponding to $[M\text{-}H]^-$ of (**5**)$_2$.

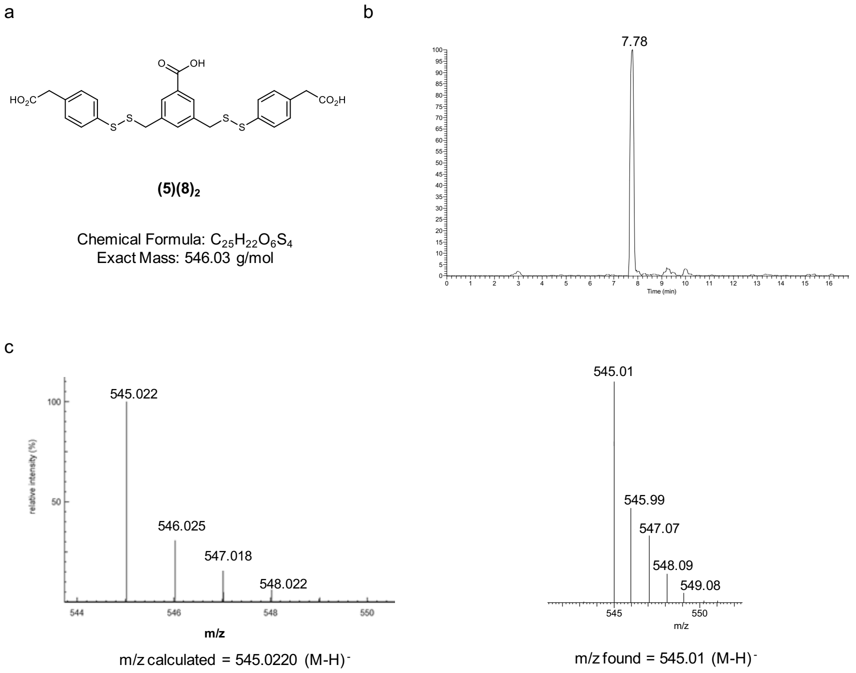

Supplementary Figure 23. **a**, Structure. **b**, Retention time analyzed by HPLC. **c**, Calculated isotope pattern and found isotope pattern corresponding to [M-H]$^-$ of (**5**)(**8**)$_2$.

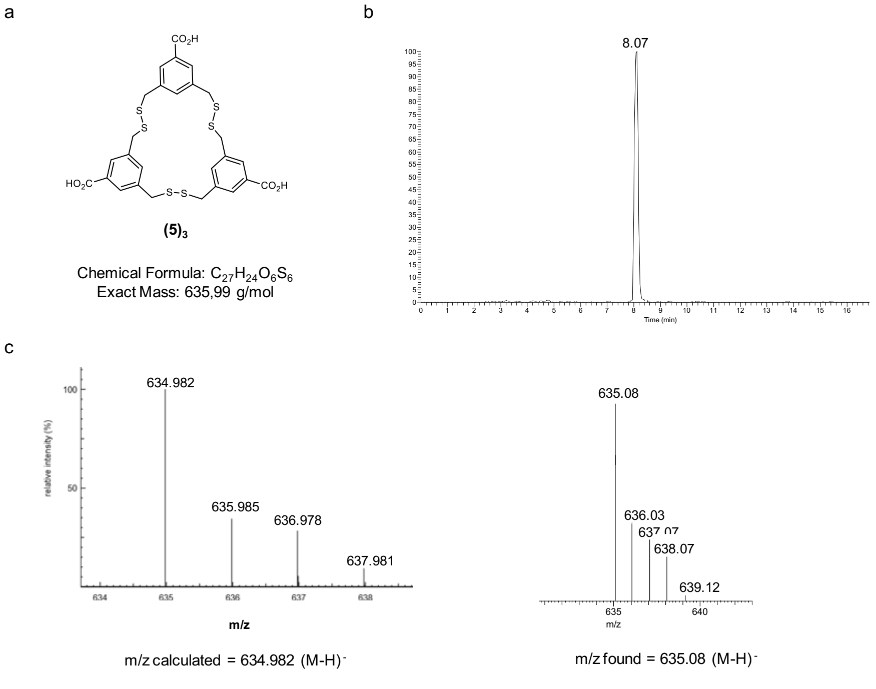

Supplementary Figure 24 **a**, Structure. **b**, Retention time analyzed by HPLC. **c**, Calculated isotope pattern and found isotope pattern corresponding to [M-H]$^-$ of (**5**)$_3$.

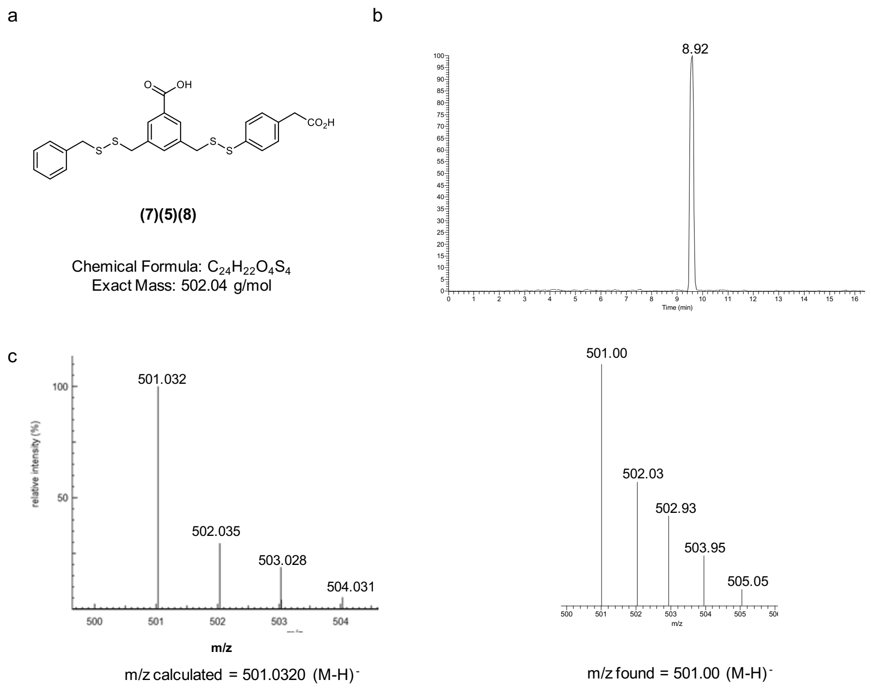

Supplementary Figure 25 a, Structure. **b**, Retention time analyzed by HPLC. **c**, Calculated isotope pattern and found isotope pattern corresponding to $[M-H]^-$ of (**7**)(**5**)(**8**).

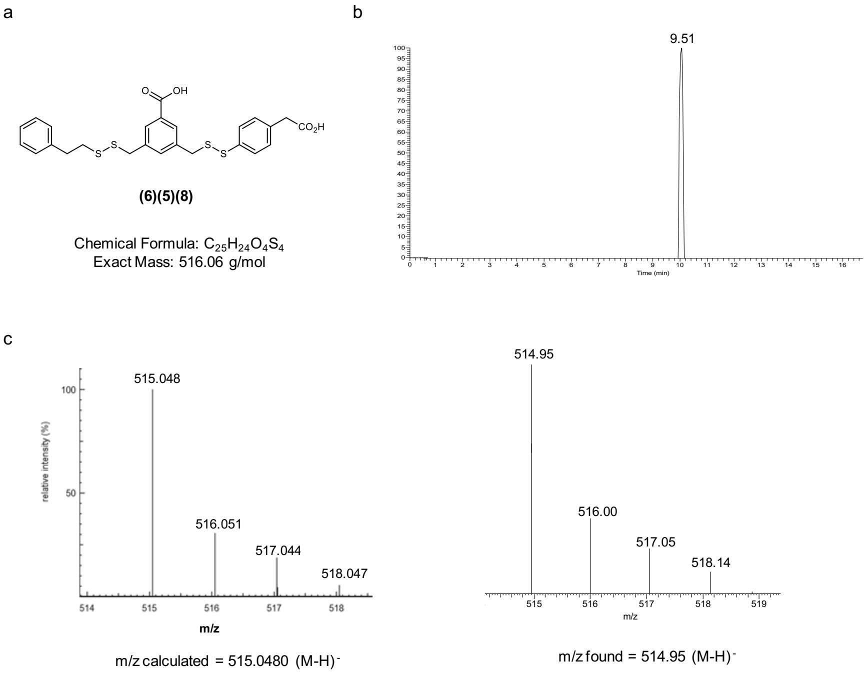

Supplementary Figure 26 a, Structure. **b**, Retention time analyzed by HPLC. **c**, Calculated isotope pattern and found isotope pattern corresponding to $[M-H]^-$ of (**6**)(**5**)(**8**).

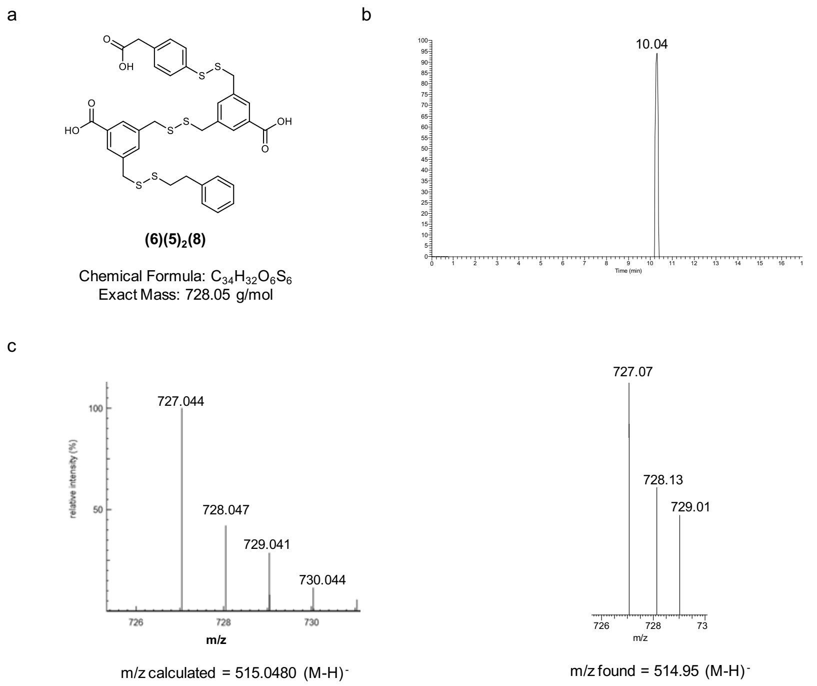

Supplementary Figure 27, a, Structure. **b**, Retention time analyzed by HPLC. **c**, Calculated isotope pattern and found isotope pattern corresponding to $[M\text{-}H]^-$ of (**6**)(**5**)$_2$(**8**).

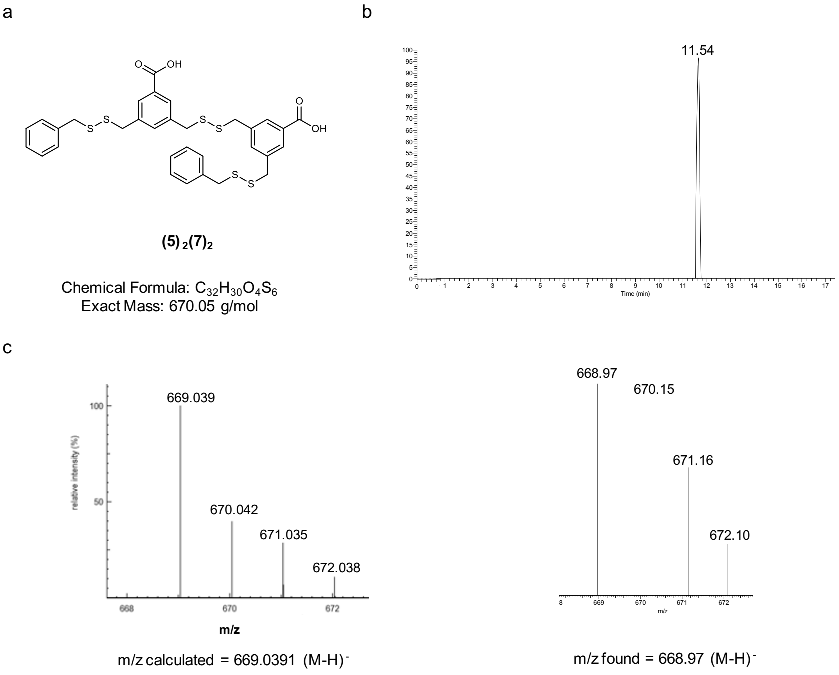

Supplementary Figure 28 a, Structure. **b**, Retention time analyzed by HPLC. **c**, Calculated isotope pattern and found isotope pattern corresponding to $[M\text{-}H]^-$ of (**5**)$_2$(**7**)$_2$.

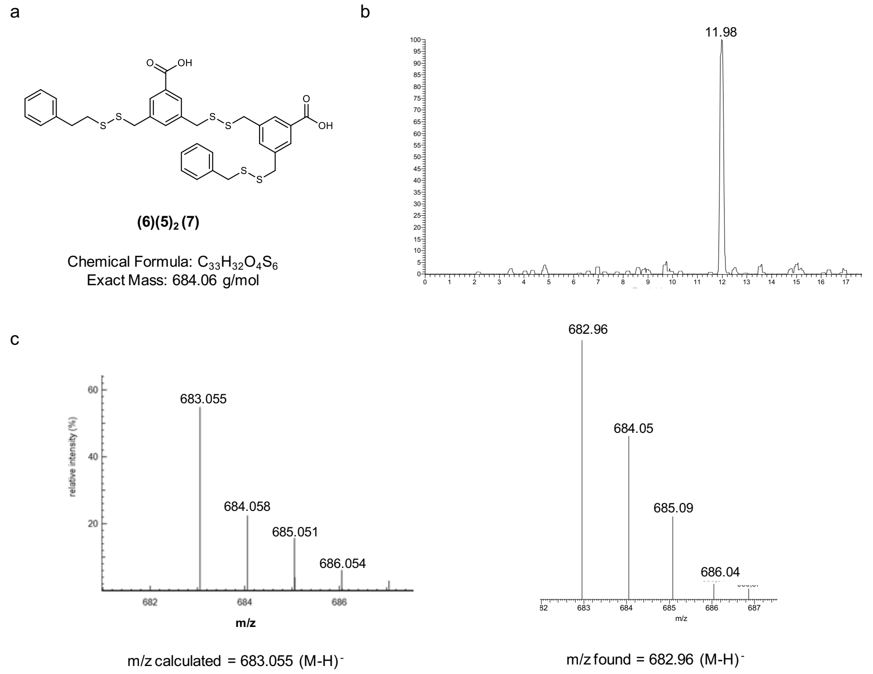

Supplementary Figure 29 a, Structure. **b**, Retention time analyzed by HPLC. **c**, Calculated isotope pattern and found isotope pattern corresponding to [M-H]$^-$ of (**6**)(**5**)$_2$(**7**).

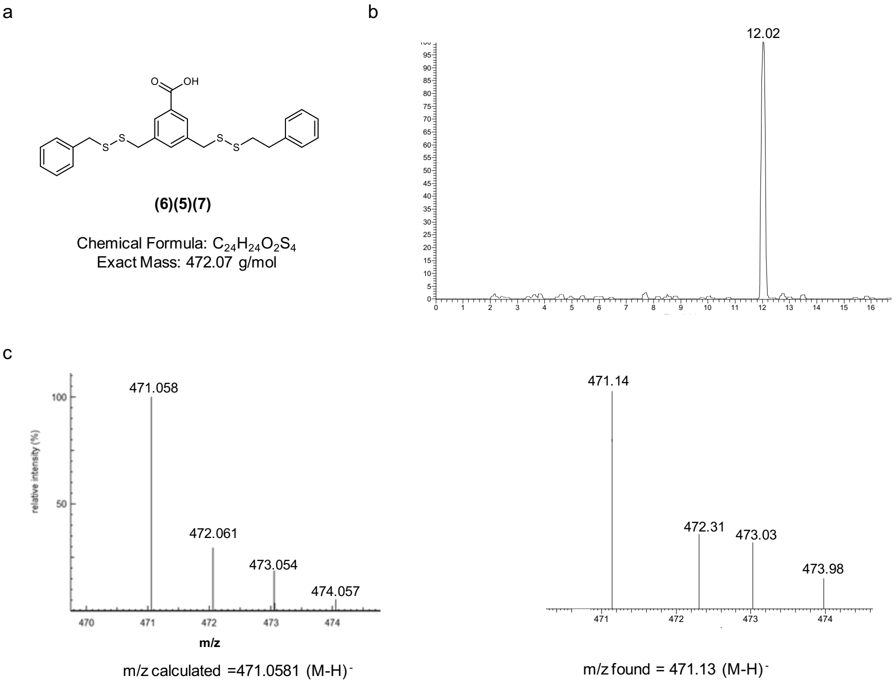

Supplementary Figure 30 a, Structure. **b**, Retention time analyzed by HPLC. **c**, Calculated isotope pattern and found isotope pattern corresponding to [M-H]$^-$ of (**6**)(**5**)(**7**).

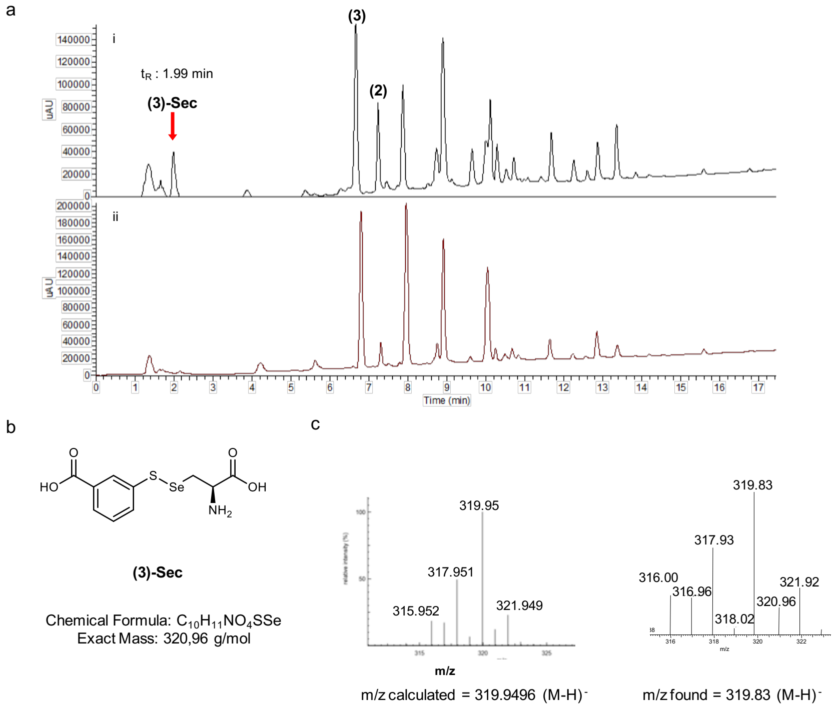

Supplementary Figure 31. (3)-Sec as selenium intermediate. **a**, Chromatograms of the DCL in presence of Sec_{ox} **(i)**, and absence of Sec_{ox}, **(ii)**, both were measured after 6 hours of equilibration where building blocks **2** and **3** were reacting. Red arrow indicates the retention time of the intermediate (**3**)**-Sec. b**, Structural information. **c**, Calculated isotope pattern and found isotope pattern corresponding to [M-H]$^-$ of (**3**)**-Sec**.

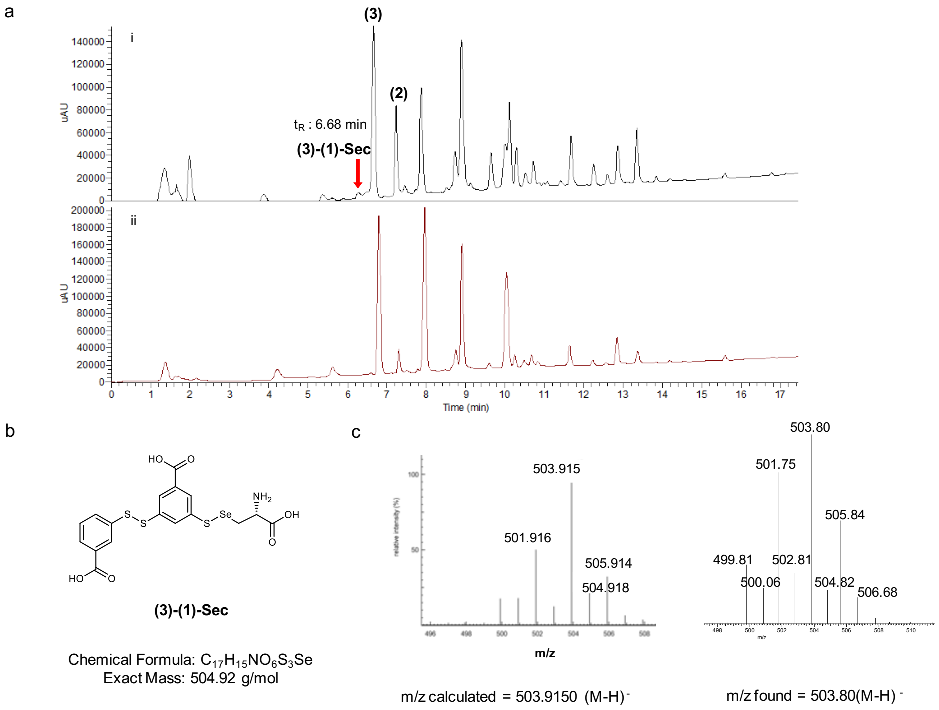

Supplementary Figure 32. (**3**)-(**1**)-Sec as a selenium intermediate. **a**, Chromatograms of the DCL in presence of Sec_{ox},(i), and absence of Sec_{ox} (ii) both were measured after 6 hours of equilibration where building blocks **2** and **3** were reacting. Red arrow indicates the retention time of the intermediate (**3**)-(**1**)-Sec**. b**, Structural information. **c**, Calculated isotope pattern and found isotope pattern corresponding to [M-H]$^-$ of (**3**)-(**1**)-Sec.

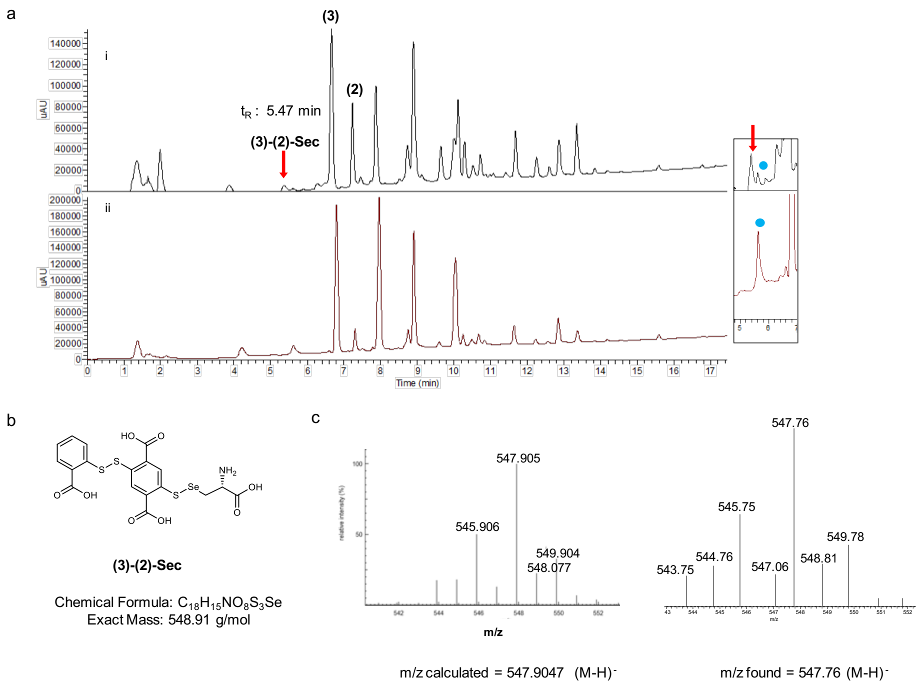

Supplementary Figure 33. (**3**)-(**2**)-Sec as a selenium intermediate. **a**, Chromatograms of the DCL in presence of Sec_{ox},(i), and absence of Sec_{ox} (ii) both were measured after 6 hours of equilibration where building blocks **2** and **3** were reacting**.** Zooming on the region of interest, the retention time of the intermediate is indicated by a red arrow whereas the blue dot shows the presence of a thiol-disulfide intermediate either catalyzed DCL (i) or blank DCL (ii). **b**, Structural information. **c**, Calculated isotope pattern and found isotope pattern corresponding to $[M\text{-}H]^-$ of (**3**)-(**2**)-Sec.

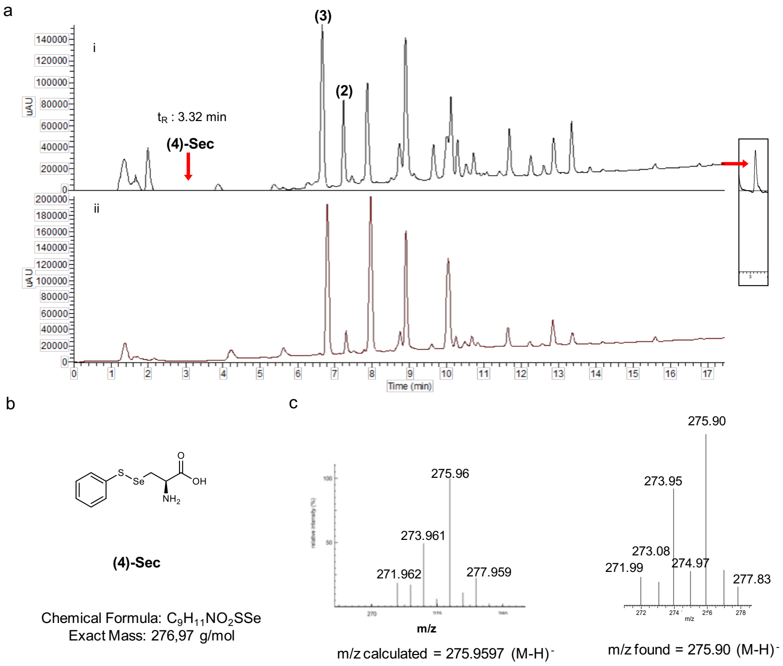

Supplementary Figure 34. (**4**)-Sec as a selenium intermediate. **a,** Chromatograms of the DCL in presence of Sec_{ox},(i) and absence of Sec_{ox} (ii) both were measured after 6 hours of equilibration where building blocks **2** and **3** were still reacting**.** Zooming on the region of interest, the retention time of the intermediate is indicated by a red arrow. **b**, Structural information. **c**, Calculated isotope pattern and found isotope pattern corresponding to $[M\text{-}H]^-$ of (**4**)-Sec.

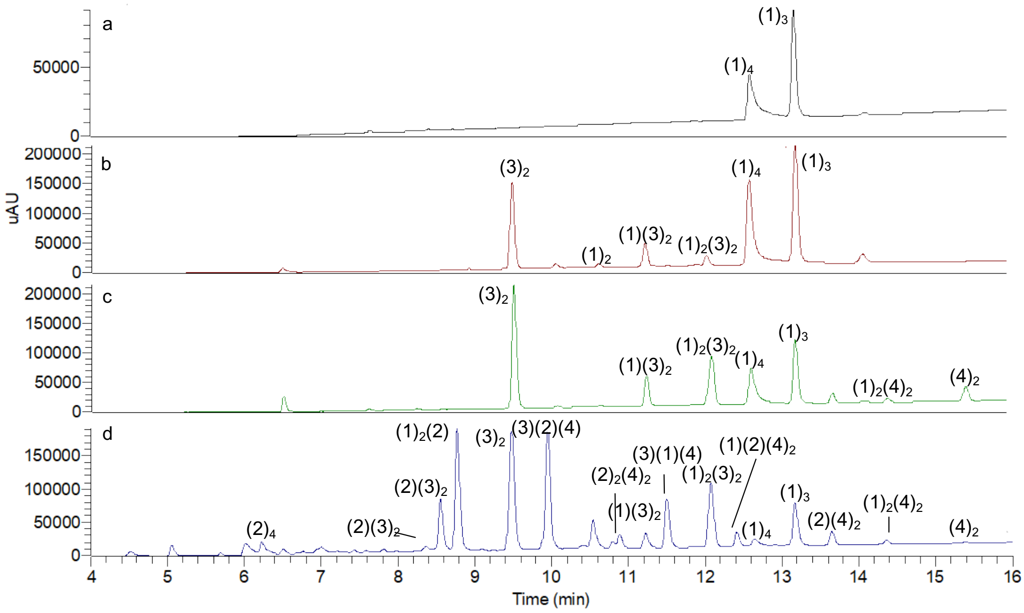

Supplementary Figure 35. Proof-of-concept for reversibility studies. The DCL was performed by the gradual addition of the thiols in presence of Sec$_{ox}$ 5% (mol). Each thiol was added once the system was previously equilibrated. The DCL was analyzed 24 hours after each addition. **a**, The starting point was the exchange of dithiol **1**. **b,** the equilibration of the thiols **1** and **3** after adding building block **3** to the system. **c,** equilibrated DCL as a result of the addition of thiol **4** to the system. **d**, after the addition of the building block **2**, the resulting chromatogram is similar to the reference DCL. Experiments were repeated three times with similar results.

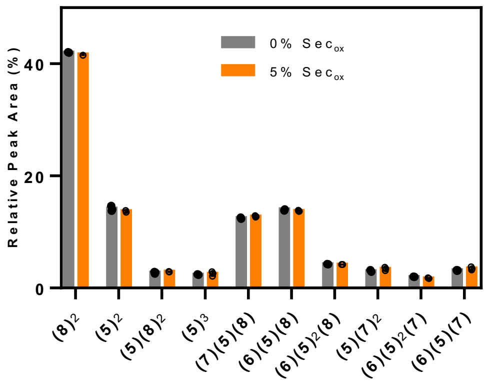

Supplementary Figure 36. Slow DCL system**.** Relative peak areas of DCLs in absence of Sec$_{ox}$ (grey bars) and presence of 5% mol Sec$_{ox}$ (orange bars). Both DCLs show a similar pattern. Mean ± SD from three independent experiments. DCL conditions: Building block (**5**) at 52,3 µM concentration and building blocks (**6-8**) at 104.6 µM concentration each. Tris buffer 20 mM pH 7.8 at 20 % (v/v) DMSO and 6ºC. See Supplementary Table 2 for data.

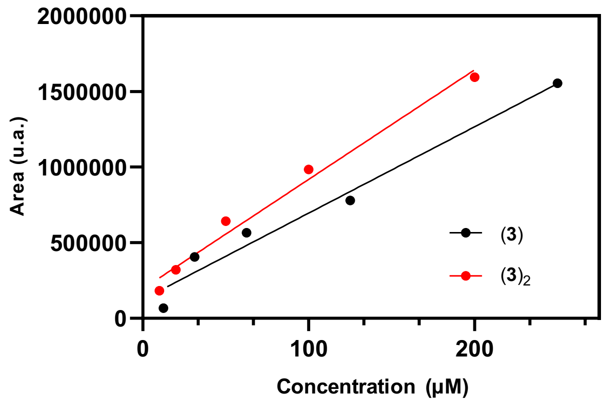

Supplementary Figure 37. Calibration curve of building block **3** and its homodimer (**3**)$_2$. Both of them were fitted to a linear least square regression. Black line is Area = 6428·[**3**]; r^2 = 0.942 and red line: Area = 8635·[(**3**)$_2$]; r^2 = 0.920. Source data are provided as Source Data file.

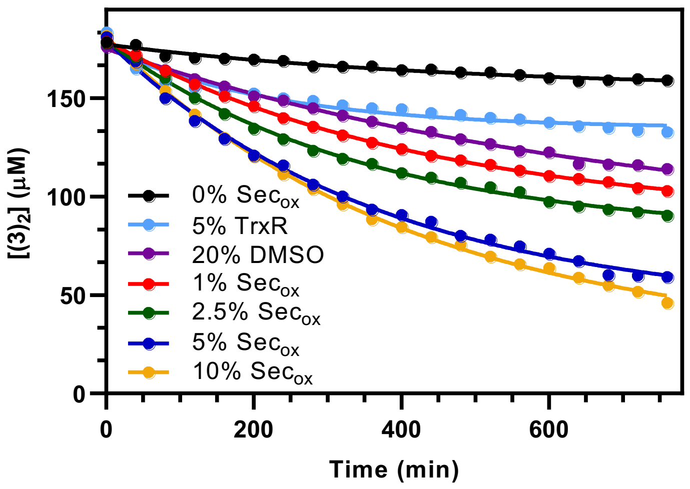

Supplementary Figure 38. Evolution of the homodimerization process from (**3**) to (**3**)$_2$ during the first 13 hours at the different conditions performed, in absence of any catalyst (black dots), in presence of TrxR at 5% mol (light blue dots), DMSO at 20% (v/v) (purple dots), Sec$_{ox}$ at 1% mol (red dots), Sec$_{ox}$ at 2.5% mol (greeen dots), Sec$_{ox}$ at 5% mol (dark blue dots), Sec$_{ox}$ at 10% mol (orange dots).The analysis of each reaction kinetic was evaluated until total completion. 0% mol Sec$_{ox}$ - 6 days, 5% mol TrxR - 5 days, 20% (v/v) DMSO - 4 days, 1% mol Sec$_{oxn}$ - 3 days, 2.5% mol Sec$_{ox}$ - 2 days, 5% mol Sec$_{ox}$ - 22 h, 10% mol Sec$_{ox}$ - 18 hours, This figure is the representation of the average values of three repetitions. Source data are provided as Source Data file.

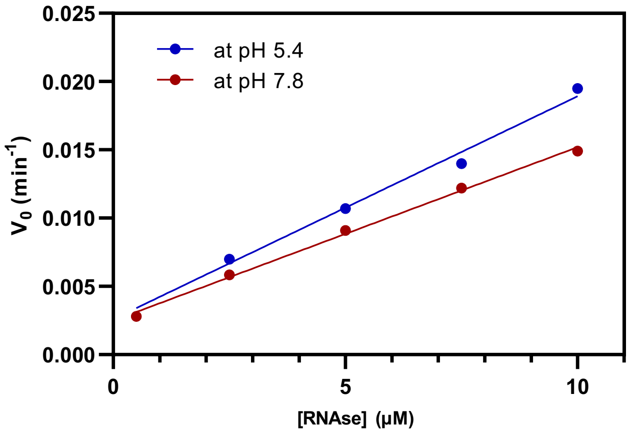

Supplementary Figure 39. Calibration curve fitting from Native RNase A at pHs 5.4 and 7.8. The data were fitted to a linear regression using a least square algorithm. At pH 5.4 (blue dots), the given equation is V_0 (min^{-1}) = 0.0016 $\mu M^{-1} \cdot min^{-1} \cdot$[RNase] (µM) + 0.0039 min^{-1}; r^2= 0.997, whereas at pH 7.8 (red dots), the given equation is V_0 (min^{-1}) = 0.0012 $\mu M^{-1} \cdot min^{-1} \cdot$[RNase] + 0.0029 min^{-1}; r^2= 0.9998. The calibration curve data were performed in triplicate. Since the withdrawn aliquot was a fixed value of 30 µL, the regression was directly performed without applying the dilution factor. Source data are provided as Source Data file.

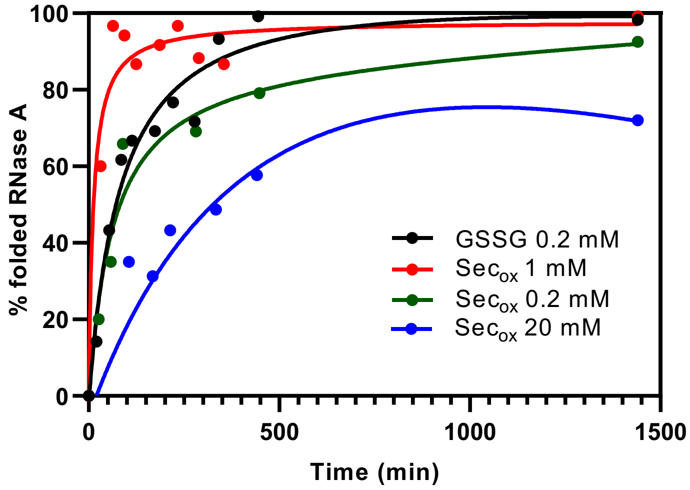

Supplementary Figure 40. Kinetic of scrambled RNase A folding at pH 7.8. Comparison of redox buffers 0.2 mM GSSG / 1 mM GSH (black dots), 1 mM Sec_{ox} / 5 mM GSH (red dots), 0.2 mM Sec_{ox} / 1 mM GSH (green dots) and 20 mM Sec_{ox} / 100 mM GSH (blue dots) in buffer 100 mM tris 2 mM EDTA pH 7.8 in presence of scrambled RNase A (5 µM) during 72 hours. 20 mM Sec_{ox} / 100 mM GSH pair shows the partial inactivation of the protein, since the large excess of diselenides supposes to be a kinetic trap to the folding. This figure is the representation of the average values of two repetitions. Source data are provided as Source Data file.

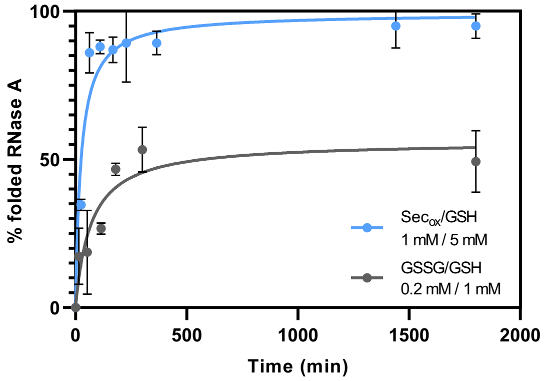

Supplementary Figure 41 Kinetic of scrambled RNase A folding at pH 5.4. Comparison of redox buffers 0.2 mM GSSG / 1 mM GSH (grey dots), 1 mM Sec$_{ox}$ / 10 mM GSH (blue dots), in buffer 100 mM PBS 2 mM EDTA pH 5.5 in presence of scrambled RNase A (5 µM) during 72 hours. Source data are provided as Source Data file. Mean ± SD from two independent experiments.

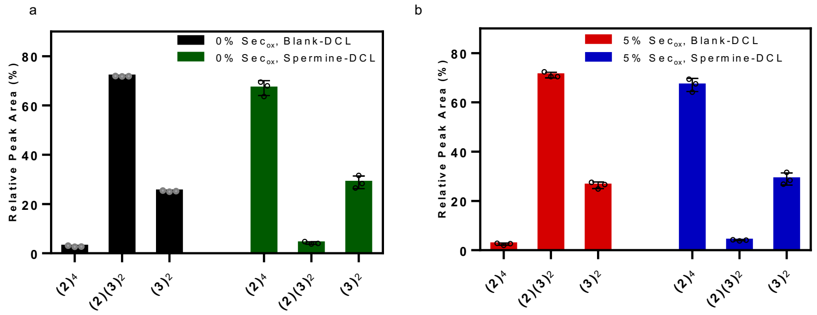

Supplementary Figure 42. Spermine templated DCL of **2** and **3**. Relative Peak Area of DCLs composed by building blocks **2** (95 µM) and **3** (190µM). They were performed in the absence of Sec$_{ox}$ and spermine at 96 h (black bars), absence of Sec$_{ox}$ and presence of spermine at 96 h (green bars), presence of 5% mol Sec$_{ox}$ and absence of spermine at 24 h (red bars), presence of 5% mol Sec$_{ox}$ and spermine at 24 h (blue bars). DCC experiments were carried out in triplicate. Mean ± SD.

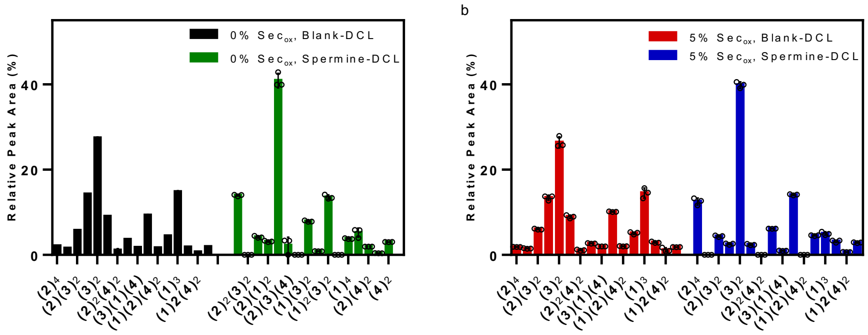

Supplementary Figure 43 Spermine templated DCL of **1-4**. RPA of the DCLs composed of building blocks **1-2** (95 µM) and **3-4** (190µM) in the absence of Sec$_{ox}$ and spermine at 96 h (black bars), absence of Sec$_{ox}$ and presence of spermine at 96 h (green bars), presence of 5% mol Sec$_{ox}$ and absence of spermine at 24 h (red bars), presence of 5% mol Sec$_{ox}$ and spermine at 24 h (blue bars). DCC experiments were carried out in triplicate. Mean ± SD.

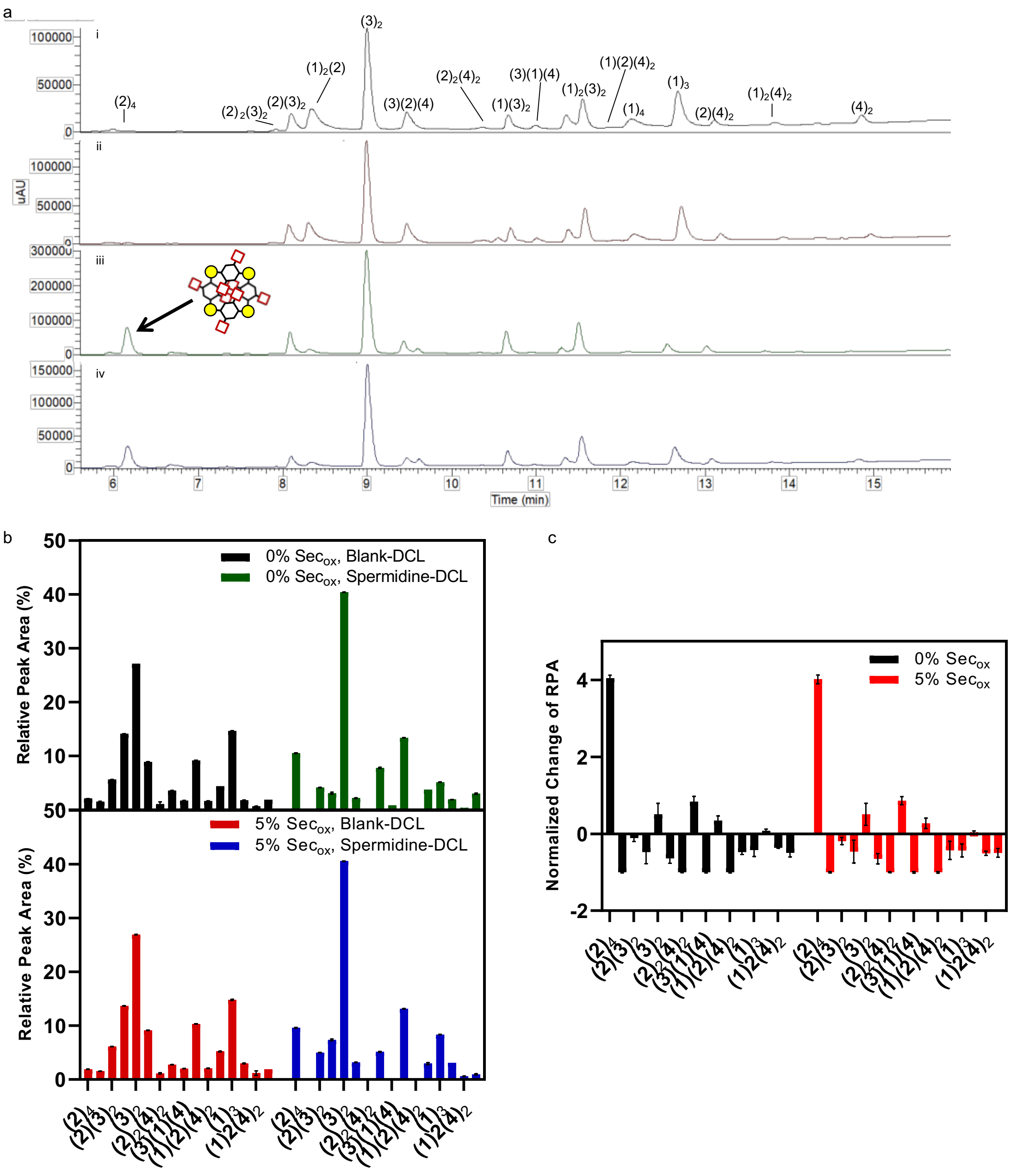

Supplementary Figure 44 Spermidine templated DCL. Yellow circle (S-S), red square (CO_2H), black hexagon (benzene ring). **a,** DCL chromatograms of building blocks **1-2**(95 µM) and **3-4** (190µM) in the absence of 5% mol Sec_{ox} and spermidine at 96 h (**i**), presence of 5% mol Sec_{ox} and absence of spermidine at 24 h (**ii**), absence of 5% mol Sec_{ox} and presence of spermidine at 96 h (**iii**), presence of 5% mol Sec_{ox} and spermidine at 24 h (**iv**). **b**, RPA of each DCL, absence of Sec_{ox} and spermidine at 96 h (black bars), absence of Sec_{ox} and presence of spermidine at 96 h (green bars), presence of Sec_{ox} and absence of spermidine (red bars), presence of Sec_{ox} and spermidine (blue bars). **c**, Normalized Change of RPA in absence (black bars) and presence of Sec_{ox} (red bars). DCC experiments were carried out in triplicate. Mean ± SD.

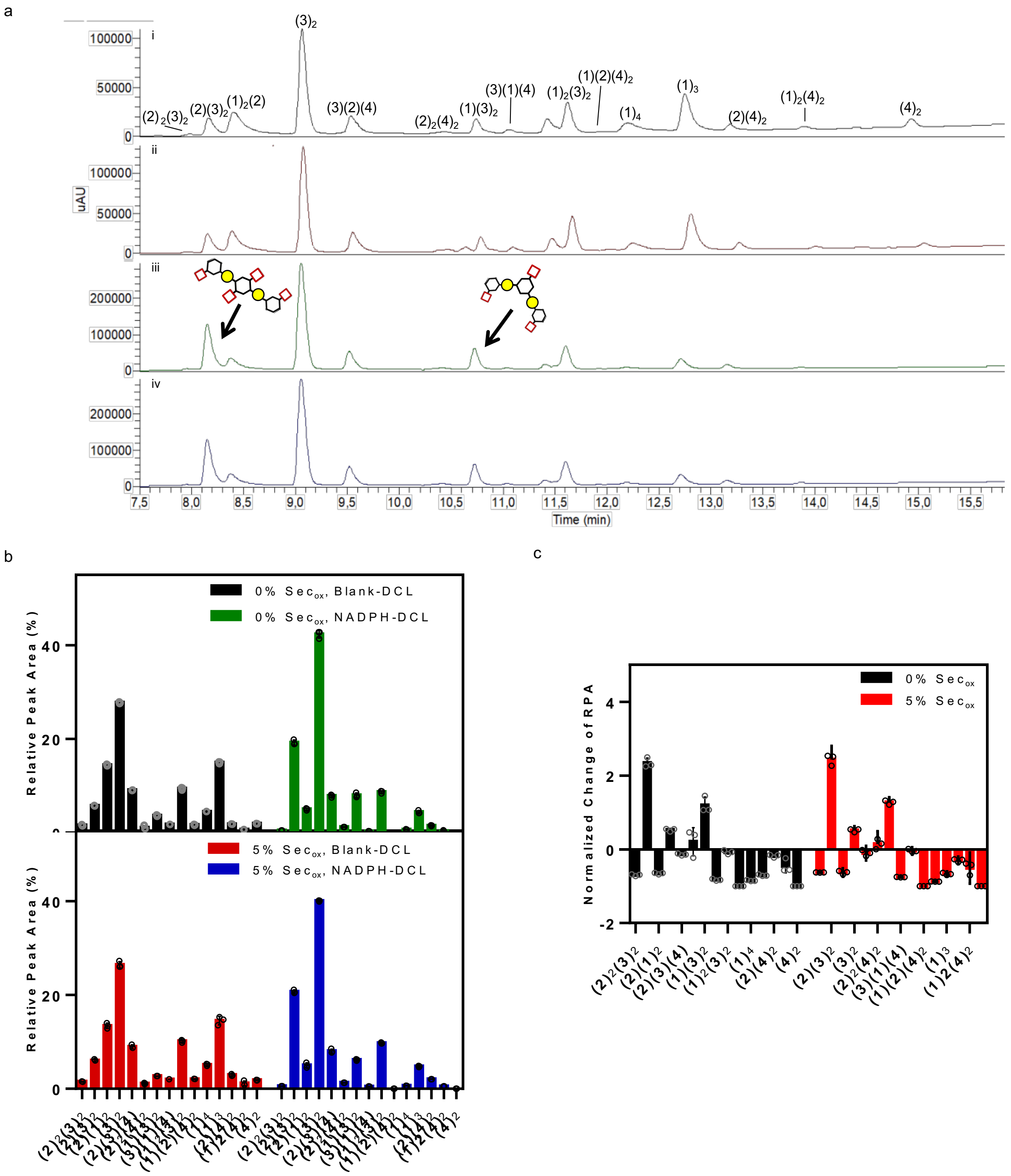

Supplementary Figure 45. NADPH templated DCL. Yellow circle (S-S), red square (CO_2H), black hexagon (benzene ring). **a,** DCL chromatograms of building blocks **1-2** (95 µM) and **3-4** (190µM) in the absence of 5% mol Sec_{ox} and NAPDH at 96 h (**i**), presence of 5% mol Sec_{ox} and absence of NADPH at 24 h (**ii**), absence of 5% mol Sec_{ox} and presence of NADPH at 96 h (**iii**), presence of 5% mol Sec_{ox} and NADPH at 24 h (**iv**). **b**, RPA of each DCL, absence of Sec_{ox} and NADPH at 96 h (black bars), absence of Sec_{ox} and presence of NADPH at 96 h (green bars), presence of Sec_{ox} and absence of NADPH (red bars), presence of Sec_{ox} and NADPH (blue bars). **c**, Normalized Change of RPA in absence (black bars) and presence of Sec_{ox} (red bars). DCC experiments were carried out in triplicate. Mean ±SD.

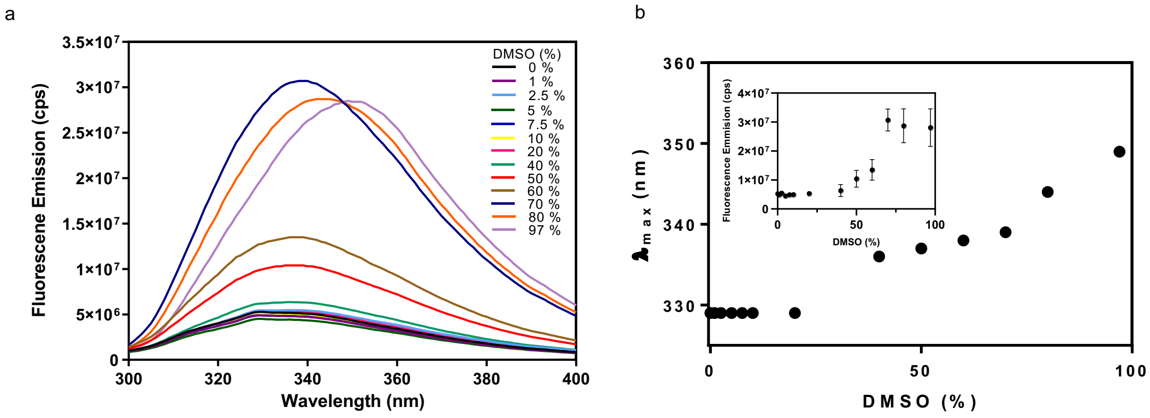

Supplementary Figure 46. GOx tolerance to DMSO (v/v). **a**, Trp emission spectra at increasing concentrations of DMSO. At 80 % (v/v) DMSO, the emission spectra maximum is decreasing due to the protein precipitation. **b**, Representation of the emission maximum (λ_{max}) observed and variation of fluorescence intensity at each DMSO concentration. Mean ± SD from four independent experiments. Source data are provided as Source Data file.

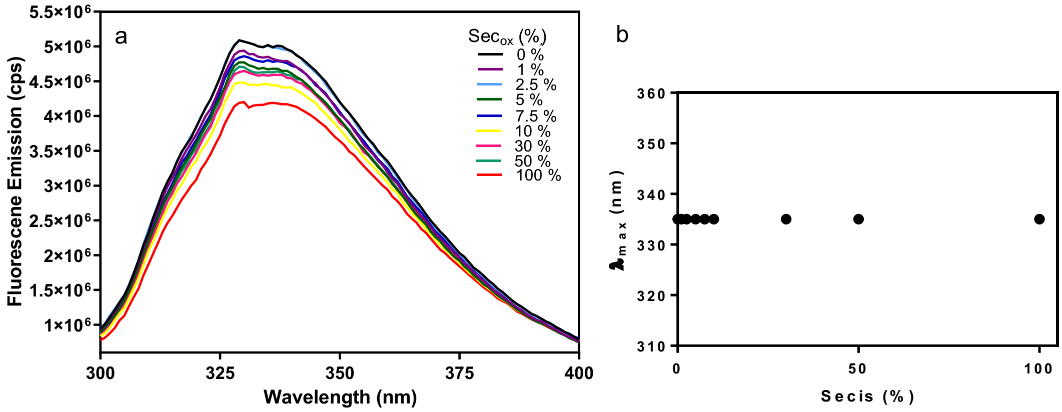

Supplementary Figure 47. GOx tolerance to Sec_{ox}. **a**, Trp emission spectra at increasing concentrations of Sec_{ox}, 100 % mol Sec_{ox} is considered as the total concentration of thiols in the DCL in presence of the protein. **b**, Representation of the emission maximum (λ_{max}) observed at each Sec_{ox} percentage (% mol). Experiments were performed in triplicate. Source data are provided as Source Data file.

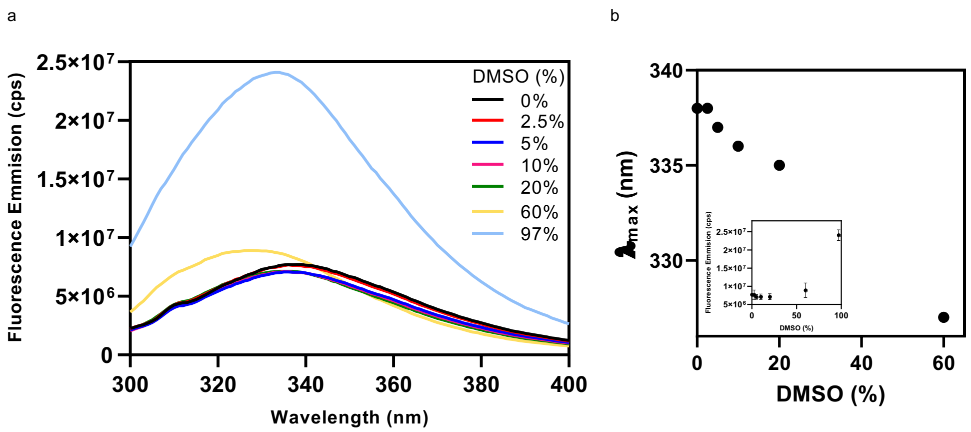

Supplementary Figure 48. BSA tolerance to DMSO. **a**, Trp emission spectra at increasing concentrations of DMSO. **b**, Representation of the emission maximum (λ_{max}) observed and variation of fluorescence intensity at each DMSO concentration. Mean ± SD from four independent experiments. Source data are provided as Source Data file.

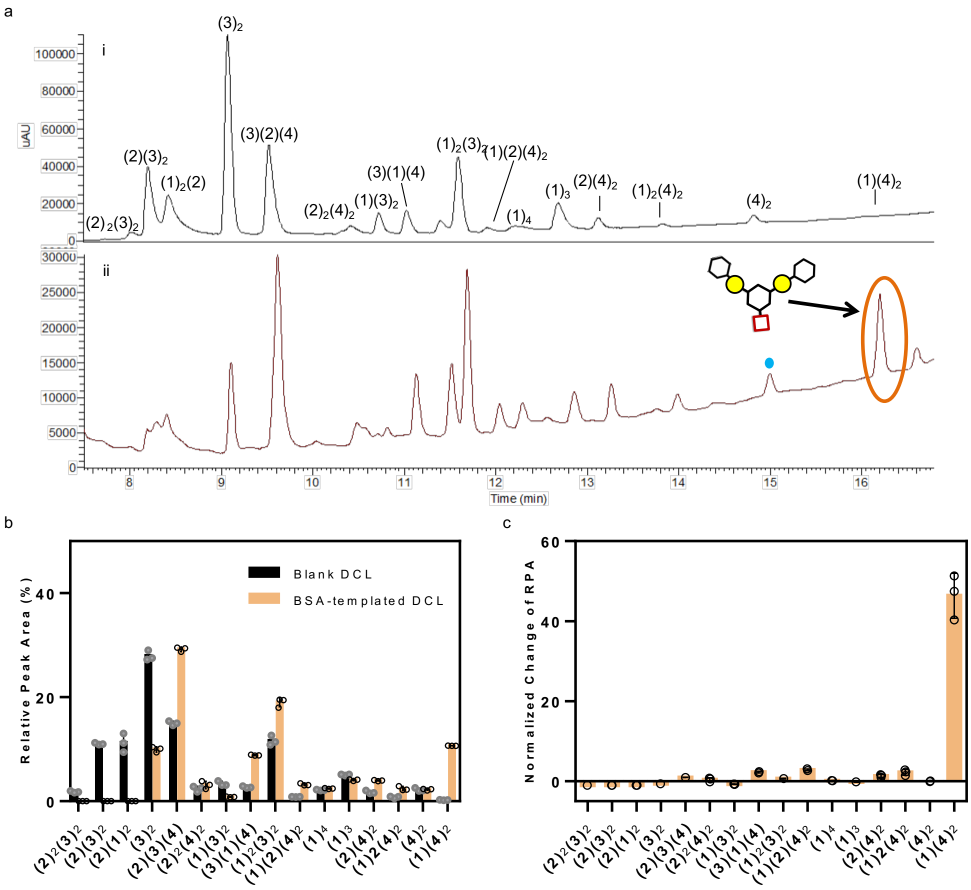

Supplementary Figure 49. BSA directed DCL. Yellow circle (S-S), red square (CO_2H), black hexagon (benzene ring). **a**, DCL chromatograms after 24 h in absence as blank (i), and presence of BSA as templated (ii). Conditions: Total thiol concentrations 1-4 (570 µM), Sec_{ox} (5% mol, 28.5 µM), BSA (57 µM), Tris buffer (20 mM, pH 7.8), T = 6 °C, 2.5 % (v/v) DMSO. **b**, BSA templated effect relative peak area (RPA) and **c**, Normalized change of RPA (see Supplementary Tables). DCC experiments were carried out in triplicate. Mean ± SD.

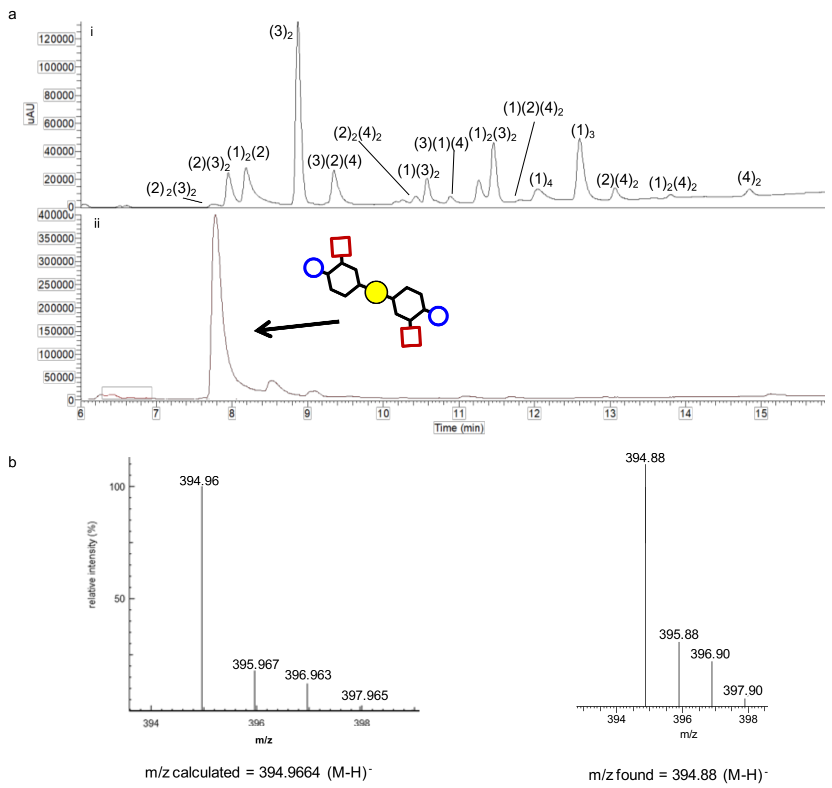

Supplementary Figure 50. GOx DCL in presence of DTNB. Yellow circle (S-S), red square (CO_2H), black hexagon (benzene ring), blue circle (NO_2). **a,** Chromatograms in absence (**i**) and presence of GOx and DTNB (1.9 mM) (**ii**). The inhibitor was added over a pre-equilibrated DCL 3 hours before treatment. Blue cirles represent NO_2 group. **b,** Calculated and found isotope pattern of the selected compound by the protein, which is DTNB. DCC experiments were carried out in triplicate. Mean ± SD.

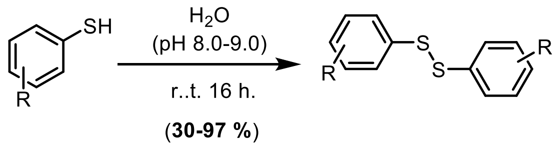

Supplementary Figure 51. General scheme of the disulfide synthesis.

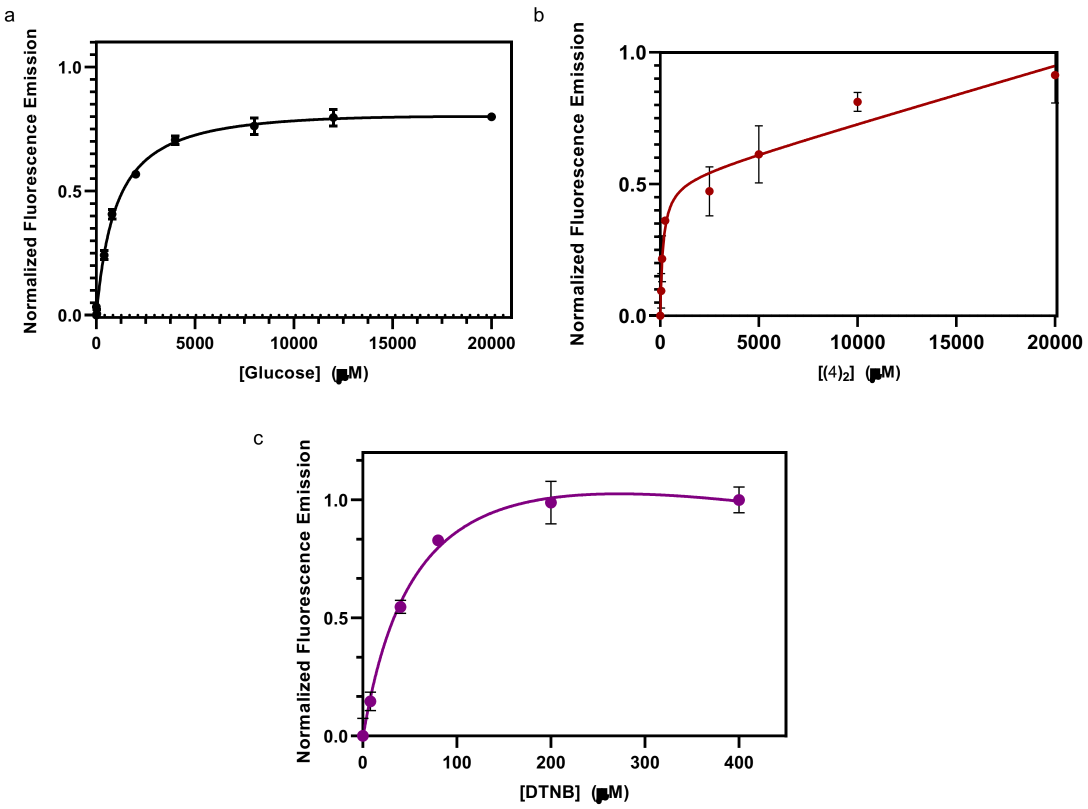

Supplementary Figure 52. Representation of the fluorescence emission of GOx at increasing concentrations of the analyzed compounds. **a**, its substrate β-D-glucose (black dots), **b**, DCL amplified inhibitor $(\mathbf{4})_2$ (red dots), **c**, the reported inhibitor DTNB (purple dots). Mean ± SD from four independent experiments. The curves represent the least squares fitting of the experimental data to one-site total model. (See Supplementary Methods and Equation). To properly compare the different curves, intensities were normalized and represented. Source data are provided as Source Data file.

2. SUPPLEMENTARY TABLES

Supplementary Table 1. Relative Peak Area (%) of each member of the DCL at increasing percentages of Sec_{ox}. Area average from each percentage is provided in Source Data File. Area is average of the three repetitions.

Compounds	$RPA_{0\%}$ (%)	$RPA_{1\%}$ (%)	$RPA_{2,5\%}$ (%)	$RPA_{5\%}$ (%)	$RPA_{10\%}$ (%)
$(2)_4$	1,83	1,82	2,09	1,92	1,85
$(2)_2(3)_2$	1,39	1,18	1,53	1,54	0,80
$(2)(3)_2$	6,32	6,26	5,64	6,14	4,94
$(1)_2(2)$	13,65	13,75	14,13	13,68	13,80
$(3)_2$	27,13	27,67	27,15	26,94	26,72
(2)(3)(4)	8,41	8,60	8,91	9,14	8,98
$(2)_2(4)_2$	0,95	0,90	1,07	1,15	1,04
$(1)(3)_2$	3,98	4,31	3,57	2,76	3,36
(3)(1)(4)	1,65	1,19	1,71	2,04	1,87
$(1)_2(3)_2$	9,25	8,83	9,20	10,33	10,51
$(1)(2)(4)_2$	1,19	1,33	1,63	2,08	1,44
$(1)_4$	3,66	4,31	4,38	5,21	4,85
$(1)_3$	15,29	14,56	14,65	14,81	14,85
$(2)(4)_2$	1,98	1,64	1,79	3,00	2,60
$(1)_2(4)_2$	0,61	1,01	0,66	1,20	1,00
$(4)_2$	2,68	2,62	1,89	1,89	1,23
$(1)(4)_2$	0,03	0,03	0,01	0,07	0,16

Supplementary Table 2. Relative Peak Area (%) of the DCL formed by building blocks **5-8**. Area is average of the three repetitions.

	0 % Sec_{ox}		5 % Sec_{ox}	
Compounds	Areas average	RPA (%)	Areas average	RPA (%)
$(8)_2$	469031	42,0	391792	41,6
$(5)_2$	158194	14,2	128647	13,7
$(5)(8)_2$	30610	2,7	27337	2,9
$(5)_3$	26429	2,4	23853	2,5
(7)(5)(8)	139031	12,4	119957	12,7
(6)(5)(8)	156777	14,0	129342	13,7
$(6)(5)_2(8)$	47382	4,2	39200	4,2
$(5)(7)_2$	33512	3,0	32037	3,4
$(6)(5)_2(7)$	21766	1,9	16073	1,7
(6)(5)(7)	35009	3,1	32655	3,5

Supplementary Table 3. Area average and Relative Peak Area (RPA) of the DCL compounds. These experiments were carried out in presence and absence of Sec_{ox} in order to demonstrate that it is compatible with biological systems, reproducing the Spermine's amplification previously reported.[2] Area is average of the three repetitions.

	Control DCL		Control-Sec_{ox} DCL	
DCL compounds	Area	RPA (%)	Area	RPA (%)
$(2)_4$	53486	2.8	50830	2.5
$(2)(3)_2$	1349590	71.9	146002	71.1
$(3)_2$	473831	25.2	1007914	26.4
	Spermine DCL		Spermine-Sec_{ox} DCL	
DCL compounds	Area	RPA (%)	Area	RPA (%)
$(2)_4$	2366156	67.2	2504710	67.0
$(2)(3)_2$	146002	4.1	151224	4.0
$(3)_2$	1007914	28.6	1083233	29.0

Supplementary Table 4. Normalized Change of RPA of Spermine DCLs.

	no Sec_{ox}	Sec_{ox}
DCL compounds	NC	NC
$(2)_4$	22.6	25.8
$(2)(3)_2$	-0.9	-0.9
$(3)_2$	0.1	0.1

Supplementary Table 5. Area average and Relative Peak Area (RPA) of the DCL compounds. These experiments were carried out in presence and absence of Sec_{ox}, reproducing the Spermine's amplification in both conditions.[2] The experiment were performed in triplicate. Area is average of the three repetitions. Sec_{ox} % is referred to % mol.

	0% Sec_{ox}, Control DCL		0% Sec_{ox}, Spermine DCL		5% Sec_{ox}, Control DCL		5% Sec_{ox}, Spermine DCL	
Compounds	Area	RPA (%)	Area	RPA (%)	Area	RPA (%)	Area	RPA (%)
$(2)_4$	31036	2,09	376013	13,81	28499	1,92	270836	12,61
$(2)_2(3)_2$	22793	1,53	0	0,00	22943	1,54	0	0,00
$(2)(3)_2$	83877	5,64	113033	4,15	91347	6,14	94862	4,42
$(1)_2(2)$	210120	14,13	84645	3,11	203479	13,68	55395	2,58
$(3)_2$	403879	27,15	1100589	40,43	400812	26,94	862059	40,15
(2)(3)(4)	132552	8,91	59888	2,20	135980	9,14	51707	2,41
$(2)_2(4)_2$	15891	1,07	0	0,00	17132	1,15	0	0,00
$(1)(3)_2$	53091	3,57	212433	7,80	41039	2,76	133003	6,19
(3)(1)(4)	25442	1,71	22903	0,84	30298	2,04	21380	1,00
$(1)_2(3)_2$	136879	9,20	365154	13,41	153654	10,33	305221	14,21
$(1)(2)(4)_2$	24256	1,63	0	0,00	30968	2,08	0	0,00
$(1)_4$	65167	4,38	102506	3,77	77534	5,21	98506	4,59
$(1)_3$	217956	14,65	140311	5,15	220320	14,81	108311	5,04
$(2)(4)_2$	26620	1,79	52608	1,93	44554	3,00	69274	3,23
$(1)_2(4)_2$	9744	0,66	10762	0,40	17783	1,20	14545	0,68
$(4)_2$	28091	1,89	81537	3,00	28091	1,89	62158	2,89

Supplementary Table 6. Normalized Change of RPA of Spermine DCLs composed by **1-4**.

Compounds	NC, 0% Sec_{ox}	NC, 5% Sec_{ox}
$(2)_4$	5,62	5,58
$(2)_2(3)_2$	-1,00	-1,00
$(2)(3)_2$	-0,26	-0,28
$(1)_2(2)$	-0,78	-0,81
$(3)_2$	0,49	0,49
(2)(3)(4)	-0,75	-0,74
$(2)_2(4)_2$	-1,00	-1,00
$(1)(3)_2$	1,19	1,25
(3)(1)(4)	-0,51	-0,51
$(1)_2(3)_2$	0,46	0,38
$(1)(2)(4)_2$	-1,00	-1,00
$(1)_4$	-0,14	-0,12
$(1)_3$	-0,65	-0,66
$(2)(4)_2$	0,08	0,08
$(1)_2(4)_2$	-0,40	-0,43
$(4)_2$	0,59	0,53

Supplementary Table 7. Area average and Relative Peak Area (RPA) of the DCL compounds. These experiments were carried out in presence and absence of Sec_{ox} casting by Spermidine.

	0% Sec_{ox}, Control DCL		0% Sec_{ox}, Spermidine DCL		5% Sec_{ox}, Control DCL		5% Sec_{ox}, Spermidine DCL	
Compounds	Area	RPA (%)	Area	RPA (%)	Area	RPA (%)	Area	RPA (%)
$(2)_4$	31036	2,09	376013	10,53	28499	1,92	270836	9,61
$(2)_2(3)_2$	22793	1,53	0	0,00	22943	1,54	0	0,00
$(2)(3)_2$	83877	5,64	113033	4,15	91347	6,14	94862	4,42
$(1)_2(2)$	210120	14,13	84645	3,11	203479	13,68	55395	2,58
$(3)_2$	403879	27,15	1100589	40,43	400812	26,94	862059	40,15
(2)(3)(4)	132552	8,91	59888	2,20	135980	9,14	51707	2,41
$(2)_2(4)_2$	15891	1,07	0	0,00	17132	1,15	0	0,00
$(1)(3)_2$	53091	3,57	212433	7,80	41039	2,76	133003	6,19
(3)(1)(4)	25442	1,71	22903	0,84	30298	2,04	21380	1,00
$(1)_2(3)_2$	136879	9,20	365154	13,41	153654	10,33	305221	14,21
$(1)(2)(4)_2$	24256	1,63	0	0,00	30968	2,08	0	0,00
$(1)_4$	65167	4,38	102506	3,77	77534	5,21	98506	4,59
$(1)_3$	217956	14,65	140311	5,15	220320	14,81	108311	5,04
$(2)(4)_2$	26620	1,79	52608	1,93	44554	3,00	69274	3,23
$(1)_2(4)_2$	9744	0,66	10762	0,40	17783	1,20	14545	0,68
$(4)_2$	28091	1,89	81537	3,00	28091	1,89	62158	2,89

Supplementary Table 8 Normalized Change of RPA of Spermidine DCLs.

Compounds	NC, 0% Sec_{ox}	NC, 5% Sec_{ox}
$(2)_4$	4,05	4,02
$(2)_2(3)_2$	-1,00	-1,00
$(2)(3)_2$	-0,11	-0,19
$(1)_2(2)$	-0,48	-0,46
$(3)_2$	0,51	0,51
(2)(3)(4)	-0,64	-0,65
$(2)_2(4)_2$	-1,00	-1,00
$(1)(3)_2$	0,83	0,86
(3)(1)(4)	-1,00	-1,00
$(1)_2(3)_2$	0,34	0,27
$(1)(2)(4)_2$	-1,00	-1,00
$(1)_4$	-0,47	-0,43
$(1)_3$	-0,42	-0,44
$(2)(4)_2$	0,06	0,04
$(1)_2(4)_2$	-0,36	-0,41
$(4)_2$	-0,49	-0,50

Supplementary Table 9. Area average and Relative Peak Area (RPA) of the NADPH-DCL compounds. These experiments were carried out in presence and absence of Sec_{ox}.

	0% Sec_{ox}, Control DCL		0% Sec_{ox}, NADPH DCL		5% Sec_{ox}, Control DCL		5% Sec_{ox}, NADPH DCL	
Compounds	Area	RPA (%)	Area	RPA (%)	Area	RPA (%)	Area	RPA (%)
$(2)_2(3)_2$	22793	1,53	17359	0,47	22943	1,54	21624	0,56
$(2)(3)_2$	83877	5,64	709562	19,24	91347	6,14	797588	20,70
$(1)_2(2)$	210120	14,13	184347	5,00	203479	13,68	192664	5,00
$(3)_2$	403879	27,15	1559899	42,31	400812	26,94	1542616	40,03
(2)(3)(4)	132552	8,91	289186	7,84	135980	9,14	310105	8,05
$(2)_2(4)_2$	15891	1,07	44457	1,21	17132	1,15	49457	1,28
$(1)(3)_2$	53091	3,57	294663	7,99	41039	2,76	237146	6,15
(3)(1)(4)	25442	1,71	11249	0,31	30298	2,04	18880	0,49
$(1)_2(3)_2$	136879	9,20	320089	8,68	153654	10,33	375247	9,74
$(1)(2)(4)_2$	24256	1,63	0	0,00	30968	2,08	0	0,00
$(1)_4$	65167	4,38	25110	0,68	77534	5,21	24803	0,64
$(1)_3$	217956	14,65	162416	4,40	220320	14,81	183891	4,77
$(2)(4)_2$	26620	1,79	55334	1,50	44554	3,00	78958	2,05
$(1)_2(4)_2$	9744	0,66	13455	0,36	17783	1,20	20304	0,53
$(4)_2$	28091	1,89	0	0,00	28091	1,89	0	0,00

Supplementary Table 10. Normalized Change of RPA of NADPH DCLs.

Compounds	NC, 0% Sec_{ox}	NC, 5% Sec_{ox}
$(2)_2(3)_2$	-0,69	-0,64
$(2)(3)_2$	2,41	2,37
$(1)_2(2)$	-0,65	-0,63
$(3)_2$	0,56	0,49
(2)(3)(4)	-0,12	-0,12
$(2)_2(4)_2$	0,13	0,11
$(1)(3)_2$	1,24	1,23
(3)(1)(4)	-0,82	-0,76
$(1)_2(3)_2$	-0,06	-0,06
$(1)(2)(4)_2$	-1,00	-1,00
$(1)_4$	-0,84	-0,88
$(1)_3$	-0,70	-0,68
$(2)(4)_2$	-0,16	-0,18
$(1)_2(4)_2$	-0,44	-0,48
$(4)_2$	-1,00	-1,00

Supplementary Table 11. Area average and Relative Peak Area (RPA) of the GOx-DCL.

Compounds	Area	RPA_{Blank} (%)	Area	RPA_{GOx} (%)
$(2)_2(3)_2$	25509	1,45	0	0,00
$(2)(3)_2$	81115	4,62	34149	7,92
$(1)_2(2)$	200637	11,42	55919	12,97
$(3)_2$	448283	25,51	71635	16,62
(2)(3)(4)	172226	9,80	28804	6,68
$(2)_2(4)_2$	29456	1,68	0	0,00
$(1)(3)_2$	68273	3,89	8971	2,08
(3)(1)(4)	53652	3,05	20212	4,69
$(1)_2(3)_2$	306590	17,45	47764	11,08
$(1)(2)(4)_2$	24206	1,38	0	0,00
$(1)_4$	55609	3,16	1959	0,45
$(1)_3$	206081	11,73	40089	9,30
$(2)(4)_2$	60691	3,45	17669	4,10
$(1)_2(4)_2$	14586	0,83	0	0,00
$(4)_2$	10391	0,59	103808	24,09

Supplementary Table 12. Normalized Change of RPA. Analysis between the blank and the GOx template DCL.

Compounds	NC of RPA
$(2)_2(3)_2$	-1,00
$(2)(3)_2$	0,72
$(1)_2(2)$	0,14
$(3)_2$	-0,35
(2)(3)(4)	-0,32
$(2)_2(4)_2$	-1,00
$(1)(3)_2$	-0,46
(3)(1)(4)	0,54
$(1)_2(3)_2$	-0,36
$(1)(2)(4)_2$	-1,00
$(1)_4$	-0,86
$(1)_3$	-0,21
$(2)(4)_2$	0,19
$(1)_2(4)_2$	-1,00
$(4)_2$	39,73

Supplementary Table 13. Area average and Relative Peak Area (RPA) of the BSA-DCL.

Compounds	Area	RPA_{Blank} (%)	Area	RPA_{BSA} (%)
$(2)_2(3)_2$	35171	1,79	0	0,00
$(2)(3)_2$	216498	11,03	0	0,00
$(1)_2(2)$	220070	11,21	0	0,00
$(3)_2$	547981	27,92	55828	9,97
(2)(3)(4)	294322	15,00	163634	29,22
$(2)_2(4)_2$	46646	2,38	17477	3,12
$(1)(3)_2$	66077	3,37	4623	0,83
(3)(1)(4)	52822	2,69	49517	8,84
$(1)_2(3)_2$	228093	11,62	106296	18,98
$(1)(2)(4)_2$	16433	0,84	18012	3,22
$(1)_4$	42020	2,14	13634	2,43
$(1)_3$	99476	5,07	22878	4,08
$(2)(4)_2$	33879	1,73	22243	3,97
$(1)_2(4)_2$	15341	0,78	13625	2,43
$(4)_2$	43143	2,20	12580	2,25
$(1)(4)_2$	4462	0,23	59721	10,66

Supplementary Table 14. Normalized Change of RPA. Analysis between the blank and the BSA-DCL.

Compounds	NC of RPA
$(2)_2(3)_2$	-1,00
$(2)(3)_2$	-1,00
$(1)_2(2)$	-1,00
$(3)_2$	-0,64
(2)(3)(4)	0,95
$(2)_2(4)_2$	0,31
$(1)(3)_2$	-0,75
(3)(1)(4)	2,28
$(1)_2(3)_2$	0,63
$(1)(2)(4)_2$	2,84
$(1)_4$	0,14
$(1)_3$	-0,19
$(2)(4)_2$	1,30
$(1)_2(4)_2$	2,11
$(4)_2$	0,02
$(1)(4)_2$	45,89

3. SUPPLEMENTARY METHODS

Materials and equipment. Most of the chemicals used in the experiments (from biological to chemistry studies) were from Sigma-Aldrich. L-Glutathione and building blocks **6** and **7** were afforded from Fluorochem**.** Building blocks **1, 2** and **5** were synthesized according to the literature.[2,3,4] Solvents were afforded from Scharlab and Panreac. Glucose Oxidase from *Aspergillus Niger*, Peroxidase from Horseradish Type II, β-D-glucose, scrambled RNASe A from bovine pancreas were purchased from Sigma Aldrich. Human TrxR2 recombinant protein was purchased from Thermofisher Invitrogen. Bovine Serum Albumin was from Roche. Melting point was measured by Büchi Melting Point M-560 device. ^{1}H-NMR and ^{13}C-NMR spectra were collected by Bruker DPX 300MHz BACS-60 equipped with QNP 5mm sounding line, operated at 300 MHz and 75 MHz. Chemical shifts are reported in ppm (δ), using the correspondent deuterated solvent. Shifts multiplicity is s: single, d: double, m: multiplet, coupling constants values (J) are measured by Hz, acquired in the Nuclear Magnetics Resonance Centre of Complutense University of Madrid. Elemental analysis was performed by LECO CHNS-932 elemental analyzer of Universidad Complutense de Madrid. Dynamic combinatorial libraries were analyzed by high-performance liquid chromatography- mass spectroscopy (HPLC-MS), in HPLC Surveyor and a Thermo Mod. FinniganTM LXQ TM Ion trap mass spectrometry system (Thermo Mod. FinniganTM LXQ TM). Solvents and formic acid were acquired from J.T. Baker and Sigma-Aldrich respectively. Analyses were performed using a reversed phase HPLC column (ACE Excel 3 C18 4.6 x 100 mm, 3 μm), using an injection volume of 25 μL, a flow rate of 1 mL/min and a gradient (20-95%) in 20 min of acetonitrile in water, both containing 0.1 % formic acid at 10 ºC. Positive ion mass spectra were acquired using electrospray ionization (drying temperature 300 ºC, sheath gas flow 60, HV capillary 6000 V, source voltage 5.50 kV, source current 100 μA). Slow DCL system was analyzed by HPLC-MS using an injection volume of 25 μL, a flow rate of 1 mL/min and a gradient (30-95%) in 17 min of acetonitrile in water, both containing 0.1 % formic acid at 10 ºC. RNase A refolding was analized by UV-vis Varian Cary 4000 spectrophotometer. Fluorescence measurements were performed by Jobin Yvon Fluorolog spectrofluorimeter equipped with Peltier thermostat. Enzymatic activity was measured spectrophotometrically by Varioskan plate reader from Thermofischer.

Synthesis of 1, 2 and **5** was performed according to the procedure described. [2, 3, 4] The characterization is described in Supplementary Discussion.

Reversibility study. The addition of each thiol was performed after the stabilization of the previous mixture. Firstly, dithiol **1** (1.8 μL, 25 mM, $4.5\cdot10^{-8}$ mol, DMSO), Sec_{ox} (1.8 μL, 7.5 mM, $1.35\cdot10^{-8}$ mol, DMSO with 4% v/v 1M NaOH) and DMSO (2.7 μL) were mixed in 20 mM Tris buffer pH 7.8 (466 μL). The DCL was analyzed by HPLC-MS after 24 hours. Monothiol **3** (1.8 μL, 50 mM, $9.0\cdot10^{-8}$ mol, DMSO) was added and analyzed after 24 hours-stabilization. Then, the addition of monothiol **4** (1.8 μL, 50 mM, $9.0\cdot10^{-8}$ mol, DMSO) was poured and the DCL was stabilized in 24 hours and analyzed by HPLC-MS. Finally, dithiol **2** (1.8 μL, 25 mM, $4.5\cdot10^{-8}$ mol, DMSO) was added. The DCL is comparable to the DCL reference. DMSO percentage is 2.5 % (v/v). Sec_{ox} percentage is 5% mol. See Supplementary Figure 35.

Kinetic studies assay. The course of the reaction was studied until the reaction finished. The kinetic analysis was performed using the first 13 hours of each reaction (See Supplementary Figure 38). The absorbance of compound **3** and its homodimer (**3**)$_2$ was measured in intervals of 40 minutes during 13 hours by HPLC (gradient from 20 to 95 % in 20 min) and then, they were controlled until reaching the complete homodimerization product. Each experiment was initiated by the addition of **3** (3.8 μL, 50 mM, $1.9\cdot10^{-7}$ mol, in DMSO) over the mixture of DMSO (17.4

μL), Sec_{ox} (3.8 μL, stock concentration according to the percentage, in DMSO with 4% v/v of 1M NaOH) or DMSO (3.8 μL) and buffer Tris 20 mM pH 7.8 (975 μL), up to a total volume of 1 mL, and a final percentage of 2.5 % v/v DMSO. Kinetic experiments were performed at 6 º C without stirring. Concentration according to the percentage of Sec_{ox}: 10 % mol – 5 mM stock ($1.9 \cdot 10^{-8}$ mol), 5 % mol – 2.5 mM stock ($9.5 \cdot 10^{-9}$ mol), 2.5 % mol – 1.25 mM ($4.75 \cdot 10^{-9}$ mol), 1% mol - 0.5 mM stock ($1.9 \cdot 10^{-9}$ mol). For the 20% (v/v) DMSO-reaction, **3** (3.8 μL, 50 mM, $1.9 \cdot 10^{-7}$ mol, in DMSO) was added over the solution of DMSO (196.2 μL) and buffer tris 20 mM pH 7.8 (800 μL) following the same parameters described before. Besides, for TrxR-catalyzed reaction, **3** (3.8 μL, 50 mM, $1.9 \cdot 10^{-7}$ mol, in DMSO)**,** TrxR (40 μL, 237.5 μM, $9.5 \cdot 10^{-9}$ mol, in buffer tris 20 mM pH 7.8), DMSO (21.2 μL) and buffer Tris 20 mM pH 7.8 (935 μL). The experiments were performed in duplicate.

To determine concentration values, a calibration curve was perform to interpolate values (Supplementary Figure 37). Then, the average data of two experiments was fitted to a second order reaction fitting, using a least square algorithm in order to obtain the K_{obs} value.

Calibration Curve for determining active RNase concentration.[5,6,7] Two calibration curves (initial velocity *vs* concentration) for acidic and basic media were performed and repeated in triplicate each. A stock solution of 50 μM Native RNase A was prepared in buffer tris 20 mM 2 mM EDTA pH 7.85 and buffer PBS 100 mM 2 mM EDTA pH 5.4. Solutions from the stocks were prepared from 0.5 to 10 μM. Aliquots (30 μL) were withdrawn to add to a freshly prepared solution of cCMP (970 μL, 450 μM) in buffer Tris 100 mM 2 mM EDTA adjusted by HCl, pH 5.9. After mixing, initial velocities were measured spectrophotometrically at 25 º C over the course of 2 min. at 292 nm.

Protein Refolding.[5,6] Refolding assay were performed at room temperature. The experiments were initiated by the addition of scrambled RNase A (100 μL, 50 μM) in a solution containing GSSG/GSH pair buffer or Sec_{ox}/GSH pair buffer. For GSSG/GSH pair, the final concentration was 5 μM scrambled RNase (100 μL, 50 μM), 0.2 mM GSSG (100 μL, 2mM), 1 mM GSH (100 μL, 10 mM) in buffer (700 μL) tris 20 mM 2 mM EDTA pH 7.85 or buffer (700 μL) PBS 100 mM 2 mM EDTA pH 5.4. For Sec_{ox}/GSH pair, different concentration pairs were prepared. At pH 7.85, 0.2 mM Sec_{ox} (100 μL, 2mM) and 1 mM GSH (100 μL, 10 mM) in buffer (700 μL) tris 20 mM pH 7.85 2 mM EDTA; 2 mM Sec_{ox} (200 μL, 10 mM) and 10 mM GSH (200 μL, 50 mM) in buffer (500 μL) tris 20 mM pH 7.85 2 mM EDTA, 20 mM Sec_{ox} (200 μL, 100 mM) and 100 mM GSH (200 μL, 500 mM) in buffer (500 μL) tris 20 mM pH 7.85 2 mM EDTA were evaluated. At pH 5.4, 1 mM Sec_{ox} (200 μL, 5 mM) and 5 mM GSH (200 μL, 25 mM) in buffer PBS 100 mM pH 5.4 2 mM EDTA was set up. All the reductant and oxidant reagents were disolved in the corresponding buffer according to the conditions. Sec_{ox} was disolved in buffer with a 10 % v/v 1M NaOH, checking that the pH. In order to determine the concentrations of folded active protein, an aliquot of 30μL was analyzed with 970 μL of 450 μM cCMP in Tris-HCl 100 mM 2 mM EDTA pH 5.9 buffer up to 1 mL at prescribed times. To determine the percentage of folded RNase, see Supplementary Equation 4.

Template-directed DCLs. Control DCL: dithiols **2-3** (2 x 1.8 μL, 25 mM, $4.5 \cdot 10^{-8}$ mol per monomer, DMSO) and monothiols **3-4** (2 x 1.8 μL, 50 mM, $9.0 \cdot 10^{-8}$ mol per monomer, DMSO), DMSO (4.5 μL) in 20 mM tris buffer pH 7.8 with 2.5 % (v/v) DMSO. It was stabilized and analyzed in 96 h at 6 º C without stirring by HPLC-MS. Control DCL Sec_{ox}: dithiols **2-3** (2 x 1.8 μL, 25 mM, $4.5 \cdot 10^{-8}$ mol per monomer, DMSO) and monothiols **3-4** (2 x 1.8 μL, 50 mM, $9.0 \cdot 10^{-8}$ mol per monomer, DMSO), Sec_{ox} (1.8 μL, 7.5 mM, $1.35 \cdot 10^{-8}$ mol, DMSO with 4% v/v 1M

NaOH), DMSO (2.7 µL) in 20 mM tris buffer pH 7.8 (466 µL) with 2.5 % v/v DMSO. Equilibration and analysis after 24 h. Template directed-DCL: dithiols **2-3** (2 x 1.8 µL, 25 mM, $4.5\cdot10^{-8}$ mol per monomer, DMSO) and monothiols **3-4** (2 x 1.8 µL, 50 mM, $9.0\cdot10^{-8}$ mol per monomer, DMSO), DMSO (4.5 µL), Spermidine or NADPH solution in 20 mM tris buffer pH 7.8 (10 µL, 2.7 mM) and 20 mM tris buffer pH 7.8 with 2.5 % (v/v) DMSO (456 µL). Analysis after stabilization in 96 hours. Template directed-Sec_{ox} catalayzed DCL: dithiols **2-3** (2 x 1.8 µL, 25 mM, $4.5\cdot10^{-8}$ mol per monomer, DMSO) and monothiols **3-4** (2 x 1.8 µL, 50 mM, $9.0\cdot10^{-8}$ mol per monomer, DMSO), Sec_{ox} (1.8 µL, 7.5 mM, $1.35\cdot10^{-8}$ mol, DMSO with 4% v/v 1M NaOH), DMSO (2.7 µL), Spermidine or NADPH solution in 20 mM tris buffer pH 7.8 (10 µL, 2.7 mM) and 20 mM tris buffer pH 7.8 with 2.5 % v/v DMSO (456 µL). DCL was analysis at 24 hours, after stabilization. Note that NADPH solution was stored in ice until adding to the DCL.

Synthesis of disulphides. A solution of the corresponding thiol (1 eq) in distilled H_2O at pH 8.0 previously adjusted with KOH 1M (60 mL) was stirred at r.t. during 16 h. Diluted HCl was added until acidic pH. The solid is washed with distilled H_2O (2 x 30 mL) by centrifugation and freeze-dried. The compounds were yielded as white powder. Yield: 30-97 %.

Protein stability experiments. In order to monitor the tolerance of GOx and BSA to DMSO, Trp emission fluorescent spectra were recorded at increasing concentrations of DMSO and under the same experimental conditions described above. For GOx, a bathochromic maximum shift was observed due to the progressive denaturalization of the protein to 350 nm. GOx tolerates up to 20% DMSO since no shift is observed at those concentrations. Therefore, the DCL has been performed in conditions where the protein is properly folded. For analyzing the GOx behavior in presence of Sec_{ox}, similar experiments were carried out. Sec_{ox} did not interfere in the GOx stability. No shift was observed in the whole range of concentrations measured. Regarding BSA, the protein tolerates up to 10 %, where no significant changes in the environment were observed. Moreover, a hyperchromic shift at increasing amounts of DMSO was expectedly observed.[8]

Fluorescence emission experiments. Tryptophan emission fluorescence of GOx (0.8 µM) was measured at increasing concentrations of substrate β-D-glucose and ligands $(4)_2$ and DTNB to determine their affinity and calculate the apparent dissociation constant, K_d'. Fluorescence experiments were carried out in buffer 20 mM Tris pH 7.8 and 5% (v/v) DMSO at 10 º C. The excitation wavelength was fixed at 295 nm, and the emission spectra range were collected over 300-400 nm. Firstly, ligands were verified not to emit in the emission range of interest. Then, four independent experiments were performed increasing GOx: ligand molar ratio from 1:0 to 1:20000 equivalents. Fluorescence intensities were normalized and represented as $(I_0 - I)/I_0$. I stands for the observed fluorescence emission at 329 nm (fluorescence maximum), and I_0 is the observed fluorescence emission of the protein itself without any small molecule.[3] The apparent dissociation constant was obtained using a least squares algorithm to fit the experimental data to a 1:1 stoichiometry model. The fitting was performed with Prism 8.3.4 using the non-linear saturation binding model for a one-total site. See Supplementary Equation 1.

$$\text{Normalized Fluorescence} = \frac{B_{max}\cdot[\text{Ligand}]}{B_{max}+K_d} \qquad [1]$$

BSA-directed DCL. Blank DCL: Dithiols **1-2** (2 x 1.8 µL, 25 mM, $4.5\cdot10^{-8}$ mol per monomer, DMSO) and monothiols **3-4** (2 x 1.8 µL, 50 mM, $9.0\cdot10^{-8}$ mol per monomer, DMSO), Sec_{ox} (1.8 µL,7.5 mM, $1.35\cdot10^{-8}$ mol, DMSO with 4% v/v 1M NaOH), DMSO (2.7 µL) in 20 mM tris buffer

pH 7.8 with 2.5 % (v/v) DMSO (466 μL). *BSA- directed DCL*: Dithiols **1-2** (2 x 1.8 μL, 25 mM, $4.5 \cdot 10^{-8}$ mol per monomer, DMSO) and monothiols **3-4** (2 x 1.8 μL, 50 mM, $9.0 \cdot 10^{-8}$ mol per monomer, DMSO), Sec_{ox} (1.8 μL,7.5 mM, $1.35 \cdot 10^{-8}$ mol, DMSO with 4% v/v 1M NaOH), DMSO (2.7 μL), BSA (135 μM, 200 μL, $2.7 \cdot 10^{-8}$ mol, 10 % mol) in 20 mM tris buffer pH 7.8 with 2.5 % v/v DMSO (266 μL). The DCL was stabilized for 24 hours at 6 º C. Then, BSA was removed by ultracentrifugation through an Amicon ultra-filter (10 KDa) and the solution was analyzed by HPLC-MS.

GOx-DCL in presence of DTNB. Blank DCL: Dithiols **1-2** (2 x 1.8 μL, 25 mM, $4.5 \cdot 10^{-8}$ mol per monomer, DMSO) and monothiols **3-4** (2 x 1.8 μL, 50 mM, $9.0 \cdot 10^{-8}$ mol per monomer, DMSO), Sec_{ox} (1.8 μL,7.5 mM, $1.35 \cdot 10^{-8}$ mol, DMSO with 4% v/v 1M NaOH), DMSO (2.7 μL) in 20 mM tris buffer pH 7.8 with 2.5 % v/v DMSO (466 μL). GOx- directed DCL: Dithiols **1-2** (2 x 1.8 μL, 25 mM, $4.5 \cdot 10^{-8}$ mol per monomer, DMSO) and monothiols **3-4** (2 x 1.8 μL, 50 mM, $9.0 \cdot 10^{-8}$ mol per monomer, DMSO), DTNB (1.8 μL, 500 mM, $9.0 \cdot 10^{-7}$ mol, DMSO), Sec_{ox} (1.8 μL,7.5 mM, $1.35 \cdot 10^{-8}$ mol, DMSO with 4% v/v 1M NaOH), DMSO (2.7 μL), GOx (135 μM, 200 μL, $2.7 \cdot 10^{-8}$ mol, 10 % mol) in 20 mM tris buffer pH 7.8 with 2.5 % v/v DMSO (266 μL). The DCL was stabilized for 24 hours at 6 º C. The addition of DTNB was done 3 hours before treatment. Then, GOx was removed by ultracentrifugation through an Amicon ultra-filter (100 KDa). HPLC analysis was perfomed.

GOx Activity Assay.[9] Enzymatic assays were perfomed by the GOx-Horseradish peroxidase coupled system with a total amount of 2.5 % (v/v) DMSO.

To elucídate the type of inhibitor, Lineweaver-Burk plot were performed. GOx activity was measured at increasing concentrations of glucose (0.2 mM to 4 mM) and in presence and absence of $(\mathbf{4})_2$ and DTNB. According to fluorescence studies and their K_d' values, the concentration of the inhibitors were chosen. For $(\mathbf{4})_2$, two concentrations were chosen: 0.7 μM (500 equivalents : 1 GOx) and 1.4 μM (1000 equivalents : 1 GOx). As DTNB owns more affinity, 70 nM (50 equivalents : 1 GOx) and 140 nM (100 equivalents : 1 GOx) were selected. The results were fitted to a non-competitive model of enzyme inbition by Prism 8.3.4. whose equation is Supplementary Equation 2.

$$\text{GOx Activity} = \frac{V'_{\text{max inh}} + [\text{glucose}]}{K_m + [\text{glucose}]}; \text{where } V'_{\text{max inh}} = \frac{V_{\text{max}}}{1 + \frac{[I]}{K_i}} \qquad [2]$$

IC_{50} was determined using different concentrations of inhibitors DTNB and $(\mathbf{4})_2$. The data collected were fitted to a model of dose-response, based on the activity against inhibitor concentration using Prism 8.3.4, whose equation is Supplementary Equation 3.

$$\text{GOx Activity} = \text{Bottom} + \frac{\text{Top} - \text{Bottom}}{1 + \frac{X}{IC_{50}}} \qquad [3]$$

4. SUPPLEMENTARY DISCUSSION

DCL members identification. The compounds ions were extracted from the TIC chromatogram. Compound amplified by GOx was isolated.

Characterization of 3,5-dimercaptobenzoic acid (1).[2,3] ^{1}H-NMR: (300 MHz, DMSO-d_6) δ 7.98 (d, J = 1.8 Hz, 2H), 7.51 (t, J = 1.8 Hz, 1H), 2.08 (s, 2H). Anal. Calcd. for $C_{14}H_{18}N_2O_4S_2$:

C, 45.14 %; H, 3.25 %; N, 0.00 %; S, 34.43 %; found: C, 44.97 %; H, 3.30 %; N, 0.05 %; S, 34.16 %. HPLC-MS: t_R: 7.02 min, $[M-H]^-$= 187/185 m/z.

Characterization of 2,5-dimercaptoterephthalic acid (2).[2,3] ^{1}H-NMR (300 MHz, DMSO-d_6): δ 8.02 (s, 2H). Anal. Calcd. for $C_{12}H_{10}S_2$: C, 41.73 %; H, 2.73%; S, 27.85; found: C, 41.51 %; H, 2.82 %; N, 0.41 %; S, 26.40. HPLC-MS: t_R: 3.9 min, $[M-H]^-$ = 231 m/z

Characterization of 3,3'-disulfanediyldibenzoic acid $(3)_2$. 3-mercaptobenzoic acid (0.62 g, 4.0 mmol). The crude was afforded as a white solid. Yield: 0.60 g, 97 %. d.p: 249-250º C. ^{1}H-NMR (300 MHz, DMSO-d_6): δ 13.15 (s, 2H), 8.02 - 7.59 (m, 2H), 7.84 – 7.66 (m, 4H), 7.47 (m, 2H).^{13}C-NMR (75 MHz, DMSO-d_6): δ 166.8 (2C), 136.7 (2C), 132.5 (2C), 131.7 (2C), 130.4 (2C), 128.9 (2C), 127.8 (2C). HRMS (m/z): $[M-H]^-$ calcd. for $C_{14}H_{10}O_4S_2$, 304.9946; found, 304.9994. Anal. Calcd, for $C_{14}H_{10}O_4S_2$: C, 54.89 %; H, 3.29 %; S, 20.93 %. Found: C, 54.81 %; H, 3.47%; S, 20.85 %. HPLC-MS: t_R: 9.70 min, $[M-H]^-$= 305 m/z.

Characterization of 1,2-diphenyldisulfane $(4)_2$. Thiophenol (500 µL, 4.88 mmol). The compound was yielded as a off-white solid (0.20 g, 30 %). m.p: 60 – 61 º C. ^{1}H-NMR (300 MHz, DMSO-d_6): δ 300 MHz, DMSO-d6) δ 7.59 – 7.48 (m, 4H), 7.47 – 7.40 (m, 2H), 7.40 – 7.24 (m, 4H). ^{13}C-NMR (75 MHz, DMSO-d_6): 136.2, 129.9 (4C), 128.1 (4C), 127.7. HRMS (m/z): $[M-H]^-$ calcd. for $C_{12}H_{10}S_2$, 217.9946; found, 217.0165 Anal. Calcd, for $C_{12}H_{10}S_2$: C, 66.02 %; H, 4.62 %; S, 29.37%. Found: C, 66.07 %; H, 4.70 %; S, 29.44 %. HPLC-MS: t_R: 15.40 min, $[M-H]^-$= 217 m/z.

Characterization of 3,5-bis(mercaptomethyl)benzoic acid (5).[4] ^{1}H-NMR (300 MHz, CD_3OD-d_4): δ 7.86 (s, 2H), 7.54 (s, 1H), 3.77 (s, 4H). HPLC-MS: t_R: min, $[M-H]^-$ = 213 m/z. HRMS: (m/z): $[M-H]^-$ calcd. for $C_9H_{10}O_2S_2$, 213.0049; found, 213.0036.

Folded RNase % calculation. The concentration of active RNase of the mixture was afforded by the calibration curve according to the conditions. Then, % of folded RNase A was obtained using the Supplementary Equation 4.

$$\%\ \text{folded RNase A} = \frac{[\text{active RNase}]_{\text{exp}}}{5\ \mu\text{M}} \text{x } 100 \qquad [4]$$

being 5 µM represents the total protein concentration.

Calculations of Relative Peak Area (RPA) and Normalized Change of RPA (NC). To analyze the influence of the protein or template in the DCL system, both parameters allow to quantify the difference between the blank DCL (in absence of any external stimuli) and in presence of the template or protein. Therefore, RPA (%) is afforded by the Supplementary Equation 5 and NC by the Supplementary Equation 6.

$$\text{RPA}\ (\%) = \frac{\text{Area}_i}{\sum_{i=\text{each peak}}^{ni} \text{Area}_i} \text{x } 100 \qquad [5]$$

$$\text{NC} = \frac{\text{RPA}_{\text{Templated}} - \text{RPA}_{\text{Blank}}}{\text{RPA}_{\text{Blank}}} \qquad [6]$$

5. SUPPLEMENTARY REFERENCES

[1] Epik, Schrödinger Suite Release 2020-2, Schrödinger LLC, New York (NY) 2020.

[2] Vial, L., Ludlow, R.F., Leclaire, J., Pérez-Fernández,, R., Otto, S. Controlling the biological effects of spermine using a synthetic receptor. *J. Am. Chem. Soc*. **128**, 10253-10257 (2006).

[3] Peter T. Corbett, P. T:; Sanders, J.K.M.; Otto, S. Exploring the relation between amplification and binding in dynamic combinatorial libraries of macrocyclic synthetic receptors in water. *Chem. Eur. J.* **14**, 2153 – 2166 (2008).

[4] Qian, Z., Rhodes, C. A., McCroskey, L. C., Wen, J., Appiah-Kubi, G., Wang, D. J., Guttridge, D.C., Pei, D. Enhancing the cell permeability and metabolic stability of peptidyl drugs by reversible bicyclization *Angew. Chem. Int. Ed.* **55**, 1-6 (2016).

[5] Beld, J., Woycechowsky, K. J., Hilvert, D. Catalysis of oxidative protein folding by small-molecule diselenides. *Biochemistry*. **47**, 6985-6987 (2008).

[6] Gough, J. D., Williams, R. H., Donofrio, A. E., Lees, W. J. Folding Disulfide-Containing Proteins Faster with an Aromatic Thiol. *J. Am. Chem. Soc.* **124 (15)**, 3885–3892 (2002).

[7] Litt, M. The kinetics of ribonuclease action on cytidine-2'-3'-cyclic phosphate. *J. Biol. Chem*. **236 (6)**, 1786-1790 (1961).

[8] Canal-Martín, A*., et al*. Insights into real-time chemical processes in a calcium sensor protein-directed dynamic library. *Nat. Comm*. **10**, *10*, 2798-2805 (2019).

[9] Pabbathi, A., Patra, S., Samanta, A. Structural transformation of bovine serum albumin induced by dimethyl sulfoxide and probed by fluorescence correlation spectroscopy and additional methods. *Chem. Phys. Chem,* **14 (11)**, 2441-2449 (2013).
